# Parameter analysis of an ovalbumin combined with aluminum hydroxide adjuvant-induced allergic rhinitis model in BALB/c mice: a systematic review and meta-analysis

**DOI:** 10.1186/s12865-026-00880-2

**Published:** 2026-07-20

**Authors:** Feng Chen, Xiaoyuan Zhou, Zhichao Niu, Ling Li, Gang Li

**Affiliations:** 1 College of Basic Medical Sciences, Yunnan University of Chinese Medicine, Kunming, China; 2 Kunming Municipal Hospital of Traditional Chinese Medicine, Kunming, China; 3 College of Chinese Materia Medica, Yunnan University of Chinese Medicine, Kunming, China; 4 Key Laboratory of Aromatic Chinese Herb Research, Yunnan Provincial University, Kunming, China

**Keywords:** Allergic rhinitis, OVA, Aluminum hydroxide, BALB/c mice, Animal model, Modeling parameters

## Abstract

**Background:**

To systematically evaluate the key modeling parameters of an allergic rhinitis (AR) model induced by ovalbumin (OVA) combined with aluminum hydroxide [Al(OH)₃] adjuvant in BALB/c mice, and to compare the effects of different sensitization and challenge protocols on model induction, thereby providing a reference for model standardization.

**Methods:**

A systematic search was conducted in PubMed, Embase, Web of Science and the Cochrane Library to identify studies using OVA combined with Al(OH)₃ to establish mouse models of AR. Data were extracted on animal strain, modeling protocols, and behavioral, immunological, and histopathological outcome measures. The model induction effects were evaluated using the standardized mean difference (SMD) and 95% confidence interval (95% CI). Subgroup analysis, meta-regression, sensitivity analysis, and the GRADE approach were performed to assess the robustness of the findings and the quality of evidence.

**Results:**

OVA combined with Al(OH)₃ effectively induced AR-related phenotypes in BALB/c mice, as evidenced by increased behavioral symptoms such as sneezing and nasal rubbing, accompanied by elevated OVA-specific IgE levels, increased expression of Th2-type inflammatory cytokines, and eosinophil infiltration in the nasal mucosa.Based on multiple outcome measures, three intraperitoneal sensitizations followed by intranasal OVA challenges for either 5 or 7 consecutive days appeared to achieve a relatively favorable model-induction effect. The effects of different OVA/Al(OH)₃ sensitization dose combinations were outcome-specific: 100 µg OVA plus 2 mg Al(OH)₃ showed a relatively large effect size for short-term sneezing responses; 50 µg OVA plus 1 mg Al(OH)₃ was more prominent in inducing eosinophil infiltration in the nasal mucosa; and 25 µg OVA plus 2 mg Al(OH)₃ exhibited a higher effect size for IL-5 levels in nasal lavage fluid. Regarding the intranasal challenge regimen, daily intranasal challenge with 400 µg OVA for either 5 or 7 consecutive days may be more favorable for inducing local Th2-type inflammatory responses in the nasal mucosa.Significant publication bias was observed for all major outcome measures, as indicated by Begg’s test (*P* < 0.001), suggesting a potential risk of overestimation of effect sizes. Nevertheless, the core conclusions remained robust after trim-and-fill correction. Some outcome measures exhibited substantial heterogeneity, which may be attributable to differences in modeling dose, administration frequency, challenge regimen, detection methods, and animal sources.

**Conclusion:**

In BALB/c mice, OVA combined with Al(OH)₃ is capable of inducing an AR model. Based on the available evidence, three intraperitoneal sensitizations followed by intranasal OVA challenges for either 5 or 7 consecutive days may serve as a basic modeling regimen for establishing an OVA/alum-induced AR model. The sensitization dose should be selected according to the primary outcome of interest: 100 µg OVA plus 2 mg Al(OH)₃ may be preferentially considered when behavioral outcomes are the main focus, whereas 50 µg OVA plus 1 mg Al(OH)₃ may be prioritized when nasal mucosal eosinophil infiltration is the primary endpoint. However, these recommendations should be interpreted with caution. The relevant evidence was mainly derived from indirect comparisons across studies, and the certainty of evidence assessed using an adapted GRADE approach was generally very low across the primary outcomes, mainly because of risk of bias, inconsistency, imprecision, and publication bias. Therefore, the above regimens should be adjusted according to the study objective, primary endpoint, and expected model intensity, rather than being interpreted as universally optimal protocols applicable to all mouse strains or experimental conditions.

**Supplementary Information:**

The online version contains supplementary material available at https://doi.org/10.1186/s12865-026-00880-2.

## Introduction

Allergic rhinitis (AR) is a common chronic inflammatory disease of the nasal mucosa that affects more than 400 million people worldwide. It substantially impairs patients’ quality of life, academic and work productivity, and imposes a considerable socioeconomic burden [1–3]. The pathogenesis and progression of AR are characterized by considerable complexity and heterogeneity, with the core mechanisms primarily involving immunoglobulin E (IgE)-mediated type I hypersensitivity reactions and T helper 2 (Th2)-type immune responses [4]. After allergens enter the nasal cavity, they first interact with nasal mucosal epithelial cells, promoting the release of epithelium-derived cytokines and activating dendritic cells. Following antigen uptake and presentation by dendritic cells, naïve T cells are driven to differentiate into Th2 cells. Th2 cells subsequently secrete cytokines such as interleukin-4 (IL-4), interleukin-5 (IL-5), and interleukin-13 (IL-13). Among these cytokines, IL-4 and IL-13 promote IgE class switching in B cells, whereas IL-5 contributes to the recruitment, activation, and survival of eosinophils [5, 6]. Specific IgE binds to high-affinity FcεRI receptors on the surface of mast cells and basophils. Upon re-exposure to the same allergen, this interaction can induce degranulation of mast cells and basophils, leading to the release of inflammatory mediators such as histamine, leukotrienes, and prostaglandins. These mediators contribute to early-phase symptoms, including sneezing, nasal itching, rhinorrhea, and nasal congestion [7, 8]. Subsequently, inflammatory cells, including eosinophils, further infiltrate the nasal mucosa, contributing to late-phase inflammatory responses and exacerbating local tissue damage.

Current clinical treatments for AR mainly include antihistamines, intranasal corticosteroids, leukotriene receptor antagonists, and allergen-specific immunotherapy. Antihistamines primarily alleviate symptoms such as nasal itching, sneezing, and rhinorrhea by blocking H1 receptors [9]; Intranasal corticosteroids can suppress inflammatory responses in the nasal mucosa and represent an important first-line treatment for moderate-to-severe AR [10, 11]; Leukotriene receptor antagonists can alleviate allergic rhinitis symptoms, such as nasal congestion, by inhibiting leukotriene-mediated inflammatory responses in the nasal mucosa [12]. In addition, allergen-specific immunotherapy can induce immune tolerance through long-term administration of standardized allergen preparations and is currently one of the therapeutic approaches with disease-modifying potential. Although current therapeutic approaches are effective in most patients, some patients still experience inadequate symptom control or insufficient responses to conventional medications [13–15]. Therefore, establishing stable and reproducible animal models of AR is essential for elucidating disease mechanisms, evaluating candidate therapeutics, and conducting pharmacodynamic and safety studies.

Animal models can simulate the sensitization, challenge, and nasal mucosal inflammatory processes of AR under controlled experimental conditions, serving as an important tool that bridges basic research and clinical translation. The model induced by ovalbumin (OVA) combined with aluminum hydroxide adjuvant [Al(OH)₃] is currently one of the most widely used classical modeling protocols. Aluminum hydroxide, as one of the most widely used aluminum-based adjuvants, enhances antigen-specific immune responses through mechanisms such as antigen adsorption, promotion of antigen uptake by antigen-presenting cells, and activation of inflammatory pathways, and it has been associated with Th2-biased immune responses. Therefore, it provides a mechanistic basis for the construction of OVA-induced allergic inflammation models [16]. OVA, one of the major protein components of egg white, is widely used as a model antigen because of its relatively high stability, well-characterized immunogenicity, and extensive application in mouse immunological studies. It is therefore commonly used in experimental research related to immune responses in mice [17]. In recent years, population-based studies have also suggested that egg white–related antigens can induce different forms of humoral and cellular immune responses, further supporting the immunological relevance of these antigens in allergic disease research [18]. OVA combined with aluminum hydroxide adjuvant can enhance antigen-specific immune responses and induce Th2-biased allergic inflammation, primarily characterized by increased levels of IL-4, IL-5, and IL-13, as well as elevated IgE production and enhanced eosinophil recruitment [19, 20], The potential significance of aluminum-related immune responses in allergic and hypersensitivity reactions in humans has also received increasing attention [21]. Therefore, the OVA combined with aluminum hydroxide adjuvant model provides an important foundation for investigating the pathogenesis of AR, evaluating the effects of pharmacological interventions, and comparing different modeling protocols. However, existing studies still vary substantially in terms of animal strain, OVA dose, aluminum hydroxide adjuvant dose, number of sensitizations, number of intranasal challenges, detection time points, and selection of outcome measures. These differences limit the comparability and reproducibility across studies and contribute to increased heterogeneity of the results. At present, systematic comparisons of the modeling parameters themselves for OVA combined with Al(OH)₃ adjuvant-induced mouse models of AR remain relatively limited. Therefore, this study used a systematic review and meta-analysis approach to comprehensively evaluate the effects of different modeling parameters on outcome measures, including nasal behavioral symptoms, total IgE, OVA-specific IgE, eosinophil infiltration, and inflammatory cytokines. This study aimed to summarize commonly used modeling protocols in the existing literature, analyze potential sources of heterogeneity, and provide a reference for the future design and standardization of mouse models of AR.

## Materials and methods

### Literature search strategy and eligibility criteria

The study was conducted in accordance with the Preferred Reporting Items for Systematic Reviews and Meta-Analyses (PRISMA) guidelines. Two authors independently searched the PubMed, Embase, Web of Science, and Cochrane Library databases from their inception to April 2026. A two-stage search strategy combining subject headings and free-text terms was used. Considering that some animal studies reported the use of aluminum hydroxide adjuvant only in the Methods section of the full text, rather than in the title or abstract, a sensitive search strategy without restrictions on aluminum hydroxide adjuvant was first applied. In the first stage, preliminary screening was performed using “allergic rhinitis,” “mouse,” and “ovalbumin” as the core search terms. In the second stage, aluminum hydroxide-related terms, including “aluminum hydroxide,” “aluminium hydroxide,” “Al(OH)₃,” and “Al(OH)3,” were added for supplementary retrieval and cross-validation.

The inclusion criteria were as follows: (1) original animal experimental studies published in English; (2) studies involving mouse models of allergic rhinitis; (3) studies using OVA as the sensitizing antigen and Al(OH)₃ as the adjuvant; (4) studies that clearly described the experimental protocol for inducing allergic rhinitis with OVA combined with Al(OH)₃ adjuvant, including key information such as the number of sensitizations, number of challenges, administration period, OVA dose, and aluminum hydroxide dose; (5) studies using intraperitoneal sensitization followed by intranasal challenge were included (6). studies that included at least a normal control group and a model group and provided extractable outcome data; and (7) studies from which at least two core model evaluation indicators could be extracted, including behavioral symptoms, IgE, OVA-specific IgE, eosinophil infiltration in the nasal mucosa, and pathological or inflammatory indicators such as IL-4 or IL-5.

The exclusion criteria were as follows: (1) reviews, conference abstracts, case reports, comments, letters, non-original studies, dissertations, or other gray literature; (2) non-animal studies, or studies in which the animal strain, grouping, or modeling protocol could not be clearly determined; (3) studies that established asthma, autoimmune disease, airway inflammation, or other disease models either alone or in combination with allergic rhinitis; (4) studies using genetically modified animals; (5) studies that reported only intervention-group results and did not provide extractable data for the normal control and model groups; and (6) studies with missing outcome data, data that could not be extracted from figures or tables, or data that remained unsuitable for quantitative analysis after reasonable processing.

### Data extraction

For studies meeting the inclusion criteria, two investigators independently extracted basic study information, baseline characteristics of the animals, model establishment protocols, outcome data, and information relevant to risk-of-bias assessment. The outcome measures included the number of sneezing episodes, frequency of nasal rubbing, serum total IgE, OVA-specific IgE, eosinophil infiltration counts in nasal tissues, and levels of inflammatory cytokines such as IL-4 and IL-5. For data that were not reported numerically in the text but were presented only in graphical form, GetData software was used to extract values according to the scales of the coordinate axes in the figures. When study results were reported as the standard error of the mean (SEM), they were converted to standard deviation (SD) using the formula SD = SEM × √n. Any discrepancies in data extraction were resolved through discussion with a third investigator. If a study included multiple assessment time points, data corresponding to the normal control group and the OVA model group and closest to the end point of model establishment were preferentially extracted. Meta-analysis was performed when at least three eligible studies were available for a given outcome; otherwise, the findings were summarized qualitatively and described narratively.

### Risk-of-bias assessment of the included studies

The risk of bias in each included study was independently assessed by two authors using SYRCLE’s risk-of-bias tool for animal studies. Each domain was judged as “Yes” indicating a low risk of bias, “No” indicating a high risk of bias, or “Unclear” indicating an unclear risk of bias. The assessed domains included random sequence generation, baseline characteristics, allocation concealment, random housing of animals, blinding of caregivers and/or investigators, random outcome assessment, blinding of outcome assessors, incomplete outcome data, selective outcome reporting, and other potential sources of bias. The final risk-of-bias assessment results were visualized using Review Manager 5.4 software.

### Statistical analysis

#### Descriptive statistical analysis

IBM SPSS Statistics software, version 27.0, was used to perform descriptive statistical analyses of modeling characteristics in the included studies, including mouse strain, number of administrations, and dosage. These variables were summarized as frequencies and percentages and ranked in descending order according to their frequency of occurrence. The results were used as a reference for subsequent subgroup analyses, meta-regression, sensitivity analyses, and parameter comparisons.

#### Effect size pooling and heterogeneity analysis

Meta-analysis was performed using Stata software, version 14.0. For continuous outcome measures, the standardized mean difference (SMD) and its 95% confidence interval (95% CI) were used as the effect size for pooled analysis. Considering the potential differences among the included studies in animal strain, modeling protocol, OVA dose, Al(OH)₃ adjuvant dose, administration frequency, and detection methods, a random-effects model was applied to pool all outcome measures. Between-study heterogeneity was assessed using Cochran’s Q test and the I² statistic. In general, an I² value of < 50% was considered to indicate low heterogeneity, whereas an I² value of ≥ 50% was considered to indicate moderate to substantial heterogeneity. When substantial heterogeneity was observed, subgroup analysis, meta-regression analysis, and leave-one-out sensitivity analysis were further conducted to explore potential sources of heterogeneity and to evaluate the robustness of the pooled results.

#### Subgroup analysis and meta-regression

To explore sources of heterogeneity and the effects of different modeling parameters on the induction efficacy of AR mouse models, subgroup analyses and meta-regression were conducted based on the SMDs of each outcome measure. Subgroup variables included the number of sensitizations, number of challenges, OVA sensitization dose, aluminum hydroxide adjuvant dose, and OVA intranasal challenge dose. For outcome measures with a sufficient number of studies, random-effects meta-regression was further performed to examine the associations between modeling parameters and effect sizes. A sufficient number of studies was defined as at least 10 independent studies for each prespecified covariate. Mouse strain was considered a potential biological effect modifier; however, because non-BALB/c strains were represented by a small and highly imbalanced number of studies, mouse strain was descriptively compared rather than included as a subgroup factor or meta-regression covariate.

#### Sensitivity analysis and assessment of publication bias

Sensitivity analysis was performed using the leave-one-out method. One study was excluded at a time, and the pooled effect size and heterogeneity statistics were recalculated to evaluate the robustness of the results. For outcome measures with substantial heterogeneity, changes in the direction of the effect size, statistical significance, and I² value before and after the sequential exclusion of individual studies were examined to determine whether any single study had a major influence on the overall results. Potential sources of heterogeneity were further discussed qualitatively. When at least 10 studies were included for a given outcome measure, funnel plots and Begg’s test were used to assess publication bias. When the number of included studies was insufficient, publication bias was described qualitatively only.

#### GRADE assessment of the certainty of evidence

The certainty of evidence for major outcomes was assessed using an adapted GRADE approach [22, 23]. Given that all included evidence was derived from preclinical animal studies rather than randomized clinical trials, the initial certainty was conservatively rated as low. Evidence certainty was further downgraded according to the main GRADE domains, including risk of bias, inconsistency, indirectness, imprecision, and publication bias.

For each outcome, we summarized the number of included studies, pooled effect estimate with 95% confidence interval, I² value, downgrading reasons, and final certainty rating. Risk of bias was judged based on methodological limitations in the included studies. Inconsistency was assessed according to statistical heterogeneity, with I² values of 50%–75% considered moderate and > 75% considered substantial heterogeneity. Indirectness was evaluated according to the consistency between the animal model, modeling procedures, outcome measures, and the review question. A limited number of studies or comparisons, small animal sample sizes, and/or wide confidence intervals were considered under imprecision. Publication bias was considered when funnel plot asymmetry or Begg’s test suggested evidence of publication bias. Because the initial certainty was rated as low and no upgrading was applied, the final certainty of evidence was classified as low or very low. Findings with low or very low certainty were interpreted cautiously.

## Results

### Literature search results and characteristics of the included studies

**Fig. 1:**
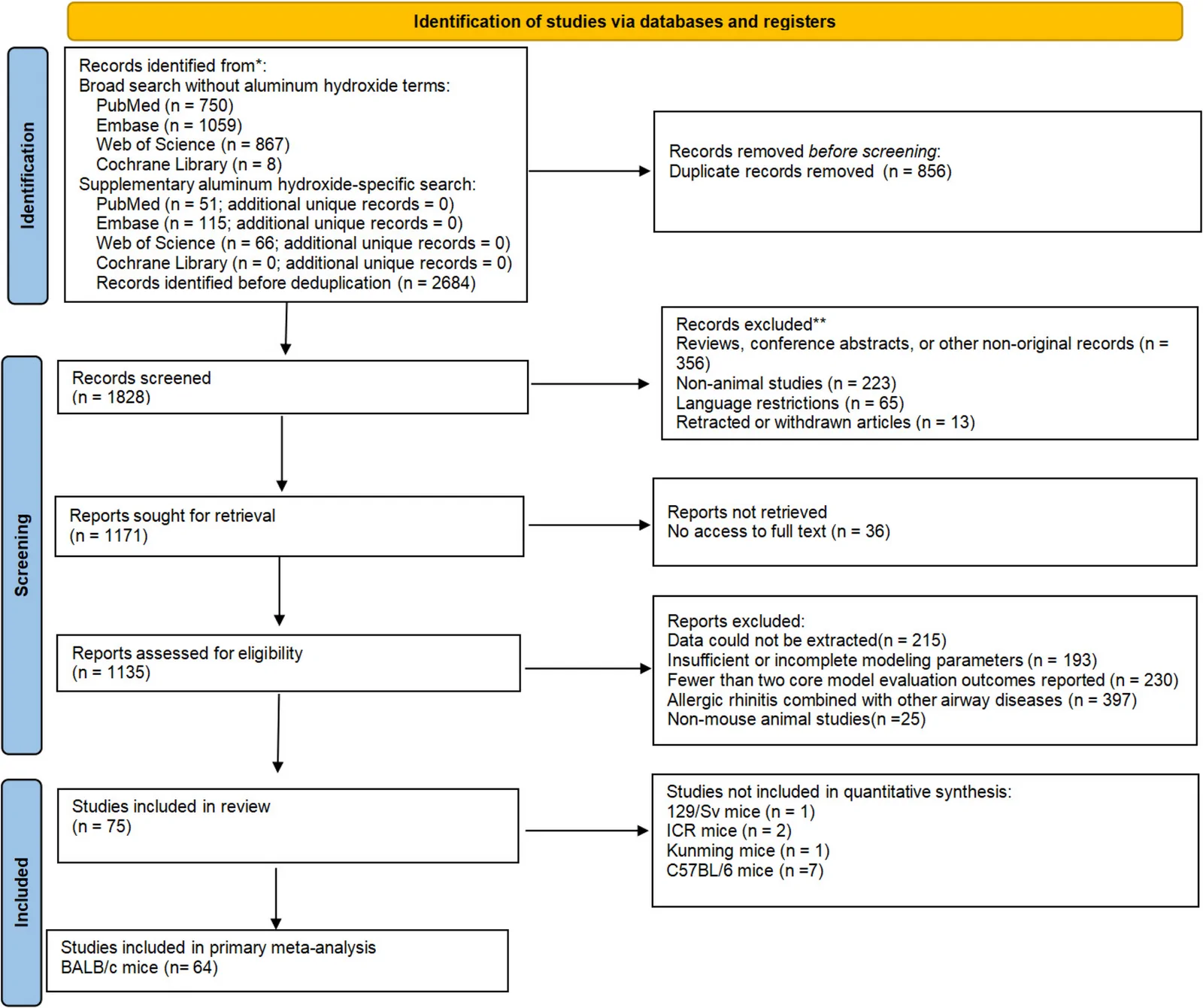
Flow diagram of the screening and selection of eligible articles

A total of 2,684 records were identified through database searches. After duplicate records were removed, 1,828 records remained for title and abstract screening. During the initial screening stage, 657 records were excluded because they were reviews, conference abstracts or other non-original studies, non-animal studies, articles excluded due to language restrictions, or retracted publications. Subsequently, 1,171 articles entered the full-text retrieval stage. Of these, 36 articles were excluded because the full texts could not be obtained, and 1,135 articles were ultimately assessed for full-text eligibility. After full-text evaluation, 1,060 articles were excluded because the data could not be extracted, modeling parameters were incomplete, core outcome measures were insufficient, rhinitis was combined with other airway diseases, or the studies involved non-mouse animal models. Finally, 75 mouse studies were included in the systematic review, among which 64 studies using BALB/c mice were included in the primary meta-analysis. The remaining studies involving C57BL/6, ICR, 129/Sv, and Kunming mice were summarized descriptively because of the limited number of studies. The detailed screening process is shown in Fig. 1.

### Risk-of-bias assessment of the included studies

**Fig. 2:**
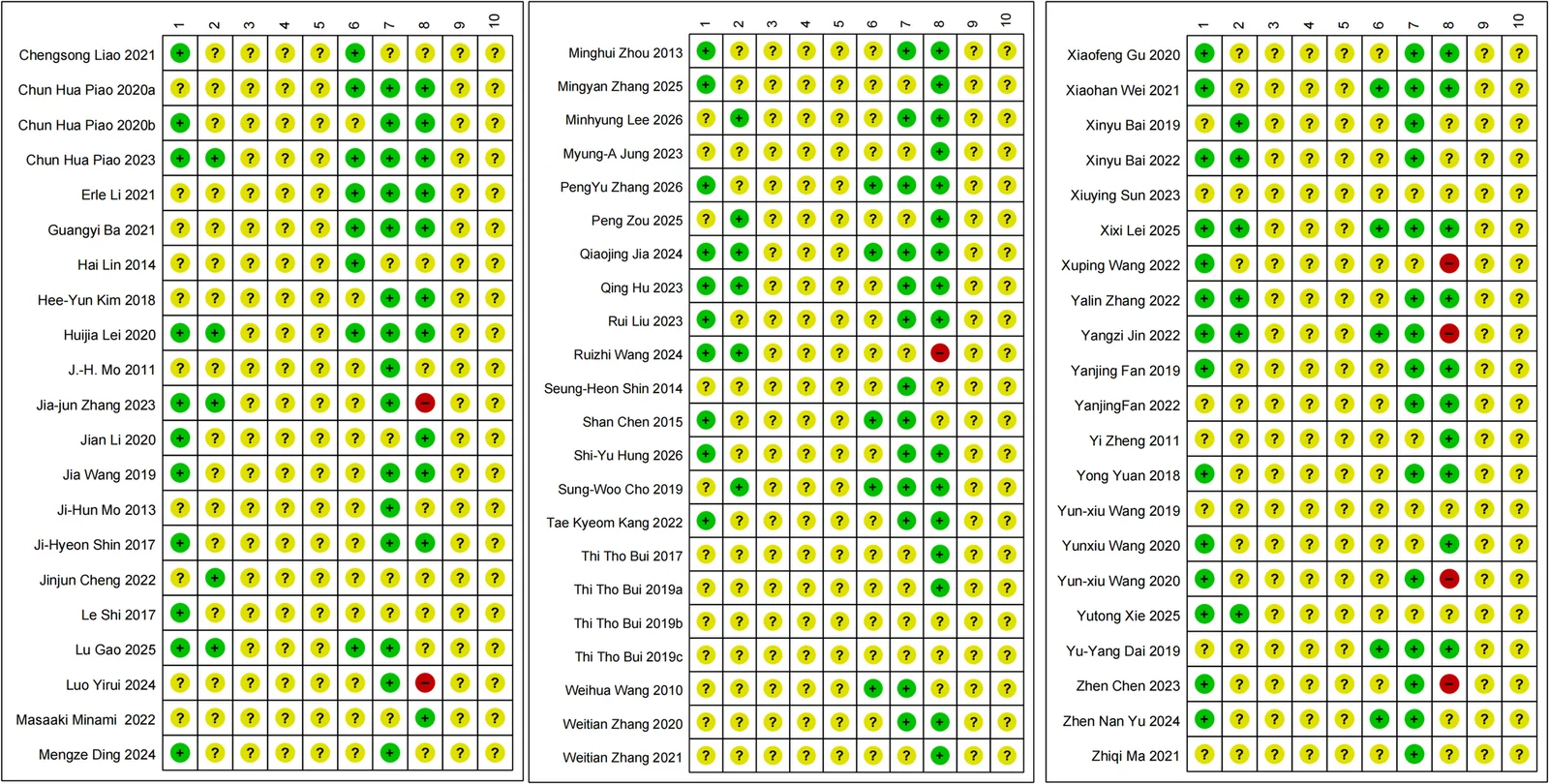
Summary of the risk-of-bias assessment of the included studies. 1, random sequence generation; 2, baseline characteristics; 3, allocation concealment; 4, random housing of animals; 5, blinding of caregivers and/or investigators; 6, random outcome assessment; 7, blinding of outcome assessors; 8, incomplete outcome data; 9, selective outcome reporting; and 10, other sources of bias. (Red indicates high risk of bias, yellow indicates unclear risk of bias, and green indicates low risk of bias)

The results of the risk-of-bias assessment are presented in Fig. 2. For different studies with the same first author and publication year, lowercase letters such as a, b, and c were added after the year or author name to distinguish them. Overall, the risk of bias in the included studies was predominantly judged as unclear. Low risk of bias was mainly observed in domains such as random sequence generation, baseline characteristics, random outcome assessment, and blinding of outcome assessors. Seventeen studies reported baseline characteristics of the experimental animals, including age, sex, and body weight, and were therefore rated as having a low risk of bias in this domain. Most studies did not adequately report information related to allocation concealment, random housing of animals, blinding of caregivers and/or investigators, selective outcome reporting, or other potential sources of bias; these domains were therefore rated as having an unclear risk of bias. Seven studies were judged to have a high risk of bias for incomplete outcome data, suggesting potential issues such as missing data, unclear handling of attrition, or incomplete reporting of results, which may have led to incomplete outcome data and affected the reliability of the findings.

### Animal strain selection, number of administrations, and dosage

#### Animal strain

Among the included mouse studies, BALB/c mice were the most commonly used strain, with a frequency of 64 studies, accounting for 85.33% of the total. This was followed by C57BL/6 mice, with a frequency of 7 studies, accounting for 9.33%. The numbers of studies using ICR, 129/Sv, and Kunming mice were relatively small, as shown in Table 1. Because the number of studies involving non-BALB/c mouse strains was limited, and because different mouse strains may differ in their immunological backgrounds and susceptibility to model induction, studies using BALB/c mice were included in the primary meta-analysis, whereas studies using other mouse strains were summarized descriptively only.

**Table 1:** Distribution of mouse strains included in the systematic review(*n* = 75)

Mouse strain	Number of studies/datasets (*n*)	Percentage(%)	Included in primary meta-analysis
BALB/c mice	64	85.33	Yes
C57BL/6 mice	7	9.33	No
ICR mice	2	2.67	No
129/Sv mice	1	1.33	No
Kunming mice	1	1.33	No
Total	75	100	—

#### Dose, frequency, and duration of administration during the sensitization phase

To improve the readability of the main text, only the major sensitization regimens used in BALB/c mouse studies are summarized in Table 2. For comparability across studies, the first day of sensitization was standardized as day 0. The sensitization protocols were classified according to the number of sensitizations, standardized sensitization time points, dosing intervals, and OVA/Al(OH)₃ dose combinations. The complete distribution of all sensitization protocol combinations, together with the corresponding statistical comparisons, is provided in Supplementary Table S2.

**Table 2:** Major sensitization regimens in BALB/c mouse studies

Number of Sensitizations	Sensitization days	OVA/Al(OH)₃ dose	Interval	*n*	%
3	0、7、14	50 µgOVA + 1 mg Al(OH)₃	Once weekly	15	23.43
3	0、7、14	25 µgOVA + 2 mg Al(OH)₃	Once weekly	9	14.06
3	0、7、14	100 µgOVA + 2 mg Al(OH)₃	Once weekly	8	12.50
3	0、7、14	100 µgOVA + 4 mg Al(OH)₃	Once weekly	6	9.38
3	0、7、14	25 µgOVA + 1 mg Al(OH)₃	Once weekly	4	6.25

Day 0 was defined as the first day of sensitization. Only major regimens are shown. The complete distribution is provided in Supplementary Table S2

The results showed that the sensitization protocols in the included studies were generally characterized by a predominant pattern of three sensitizations administered once weekly, with the most common standardized sensitization time points being days 0, 7, and 14. Regarding specific dose combinations, the most frequently used regimen was 50 µg OVA plus 1 mg Al(OH)₃, which was reported in 15 studies and accounted for 23.43% of the total. This was followed by 25 µg OVA plus 2 mg Al(OH)₃, accounting for 14.06%; 100 µg OVA plus 2 mg Al(OH)₃, accounting for 12.50%; 100 µg OVA plus 4 mg Al(OH)₃, accounting for 9.38%; and 25 µg OVA plus 1 mg Al(OH)₃, accounting for 6.25%.

These findings indicate that, in BALB/c mouse models of allergic rhinitis induced by OVA combined with Al(OH)₃, the sensitization phase has developed a relatively concentrated dosing schedule, with three systemic sensitizations commonly performed on days 0, 7, and 14. However, substantial variation remains in the combinations of OVA antigen dose and Al(OH)₃ adjuvant dose. Because antigen and adjuvant doses may influence the magnitude of Th2-type immune response induction, IgE production, and subsequent nasal inflammatory responses, differences in sensitization dose combinations may represent an important source of heterogeneity among the included studies.

#### Dose, frequency, and duration of administration during the challenge phase

To characterize the challenge protocols used in BALB/c mouse studies, the first day of intranasal challenge was standardized as day 0. The protocols were classified according to the number of challenges, challenge days, daily OVA dose, and dosing interval. Table 3 presents the major challenge regimens, whereas the complete distribution is provided in Supplementary Table S3.

**Table 3:** Major challenge regimens in BALB/c mouse studies

Number of Challenges	Challenge days	OVA dose	Interval	*n*	%
7	Days 0–6	100 µgOVA/d	Once daily	8	12.50
8	Days 0–7	800µgOVA/d	Once daily	6	9.38
7	Days 0–6	400µgOVA/d	Once daily	5	7.81
7	Days 0–6	500µgOVA/d	Once daily	5	7.81

Day 0 was defined as the first day of challenge. Only major regimens are shown. The complete distribution is provided in Supplementary Table S3

The results showed that the challenge phase in the included studies was generally characterized by continuous intranasal challenge as the predominant pattern. The number of challenges was most commonly concentrated at 7 or 8 times, with standardized challenge time points mainly on days 0–6 or days 0–7, and once-daily administration was the most frequently used dosing interval. Regarding the distribution of specific protocols, the most frequently used regimen was continuous challenge seven times from days 0 to 6 with a daily OVA challenge dose of 100 µg/day, which was adopted in 8 studies and accounted for 12.50% of the total. This was followed by continuous challenge eight times from days 0 to 7 with a daily OVA challenge dose of 800 µg/day, accounting for 9.38%. Regimens involving continuous challenge seven times from days 0 to 6 with daily OVA challenge doses of 400 µg/day and 500 µg/day each accounted for 7.81%.

These findings indicate that intranasal challenge protocols in OVA-induced BALB/c mouse models of allergic rhinitis were relatively concentrated in terms of dosing frequency and scheduling, with most studies using continuous daily challenge to simulate repeated antigen exposure of the nasal mucosa. However, substantial differences remained among studies in the daily OVA challenge dose and cumulative challenge exposure. Because the intranasal challenge phase directly determines the intensity of local antigen stimulation in the nasal mucosa and further affects behavioral manifestations such as sneezing, nasal scratching, or nasal rubbing, as well as inflammatory cell infiltration in the nasal mucosa, Th2-type cytokine expression, and histopathological changes, differences in challenge dose and challenge frequency may represent important sources of variation in the phenotypic severity of AR mouse models and heterogeneity across studies.

### Meta-analysis results

#### Behavioral symptoms

To avoid potential measurement bias caused by combining different observation periods, random-effects meta-analyses were performed separately for the numbers of sneezing episodes and nasal scratching or rubbing events recorded within 10, 15, and 20 min. The results showed that, compared with the normal and modle groups, the model groups induced by OVA combined with Al(OH)₃ exhibited more pronounced nasal behavioral responses across different observation time windows.

**Fig. 3:**
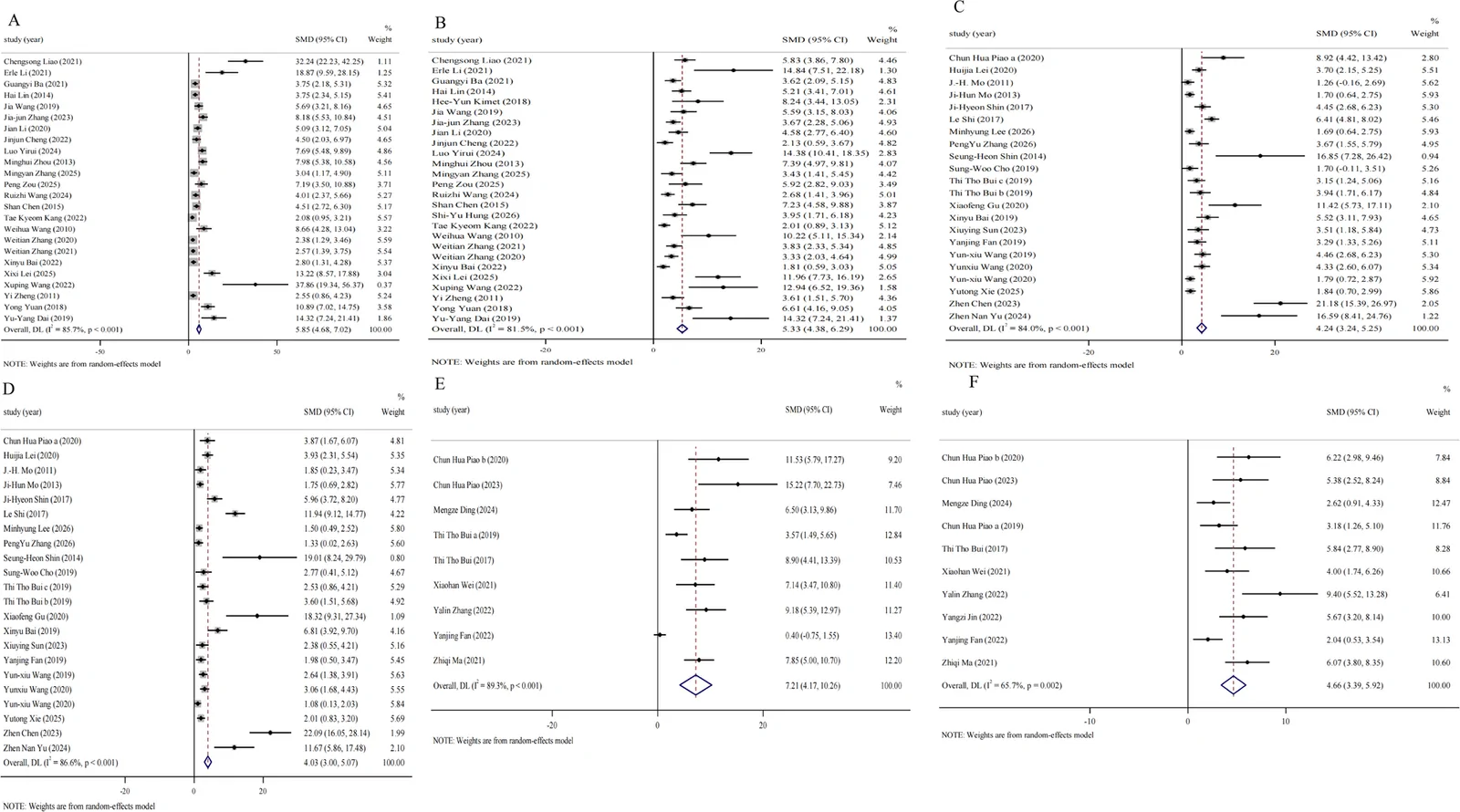
Meta-analysis results of nasal behavioral symptoms in OVA/Al(OH)₃-induced AR model mice across different observation periods. **A**, Number of sneezing episodes within 10 min; **B**, number of nasal scratching or rubbing events within 10 min; **C**, number of sneezing episodes within 15 min; **D**, number of nasal scratching or rubbing events within 15 min; **E**, number of sneezing episodes within 20 min; **F**, number of nasal scratching or rubbing events within 20 min. (Note: Effect sizes are presented as SMDs with 95% confidence intervals (95% CIs), and pooled analyses were performed using a random-effects model. Squares represent the effect sizes of individual studies, with the size of each square indicating the study weight; horizontal lines represent 95% CIs; and diamonds represent the pooled effect sizes)

For the numbers of sneezing episodes and nasal scratching or rubbing events within 10 min, the pooled effect sizes were SMD = 5.85 (95% CI: 4.68–7.02, I² = 85.7%) and SMD = 5.33 (95% CI: 4.38–6.29, I² = 81.5%), respectively. For the numbers of sneezing episodes and nasal scratching or rubbing events within 15 min, the pooled effect sizes were SMD = 4.24 (95% CI: 3.24–5.25, I² = 84.0%) and SMD = 4.03 (95% CI: 3.00–5.07, I² = 86.6%), respectively. For the numbers of sneezing episodes and nasal scratching or rubbing events within 20 min, the pooled effect sizes were SMD = 7.21 (95% CI: 4.17–10.26, I² = 89.3%) and SMD = 4.66 (95% CI: 3.39–5.92, I² = 65.7%), respectively, as shown in Fig. 3.

These findings indicate that the BALB/c mouse model of AR established using OVA combined with Al(OH)₃ exhibited a marked increase in nasal symptoms at the behavioral level, suggesting that this model can effectively simulate AR-related nasal responses. However, moderate to high heterogeneity was observed across all behavioral outcomes, indicating that differences among studies in sensitization protocols, challenge protocols, OVA dose, observation time windows, behavioral recording methods, and investigator-based assessment criteria may have influenced the magnitude of the pooled effect sizes.

#### IgE and OVA-specific IgE

**Fig. 4:**
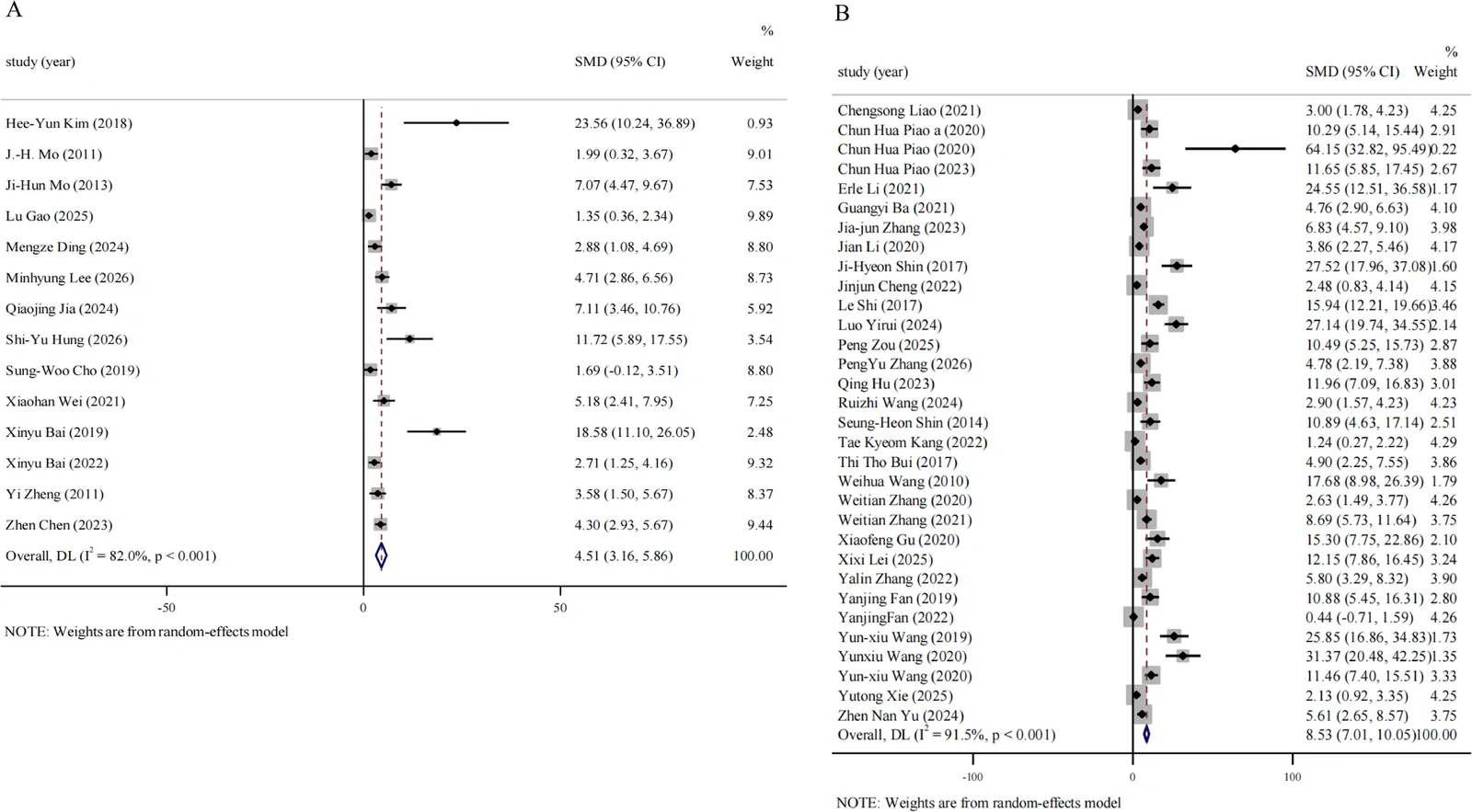
Meta-analysis results of serum IgE-related immunological indicators in OVA/Al(OH)₃-induced AR model mice. **A**, Serum total IgE level; **B**, serum OVA-specific IgE level. (Note: Effect sizes are presented as SMDs with 95% CIs, and pooled analyses were performed using a random-effects model. Squares represent the effect sizes of individual studies, with the size of each square indicating the study weight; horizontal lines represent 95% CIs; and diamonds represent the pooled effect sizes)

To evaluate the systemic humoral immune characteristics of the BALB/c mouse AR model induced by OVA combined with Al(OH)₃, random-effects meta-analyses were performed separately for serum total IgE and OVA-specific IgE. The results showed that, compared with the normal control group, the model group had significantly increased total IgE levels, with a pooled effect size of SMD = 4.51 (95% CI: 3.16–5.86, *P* < 0.001) and I² = 82.0%. OVA-specific IgE was also significantly increased, with a pooled effect size of SMD = 8.53 (95% CI: 7.01–10.05, *P* < 0.001) and I² = 91.5%, as shown in Fig. 4. These findings indicate that OVA combined with Al(OH)₃ modeling significantly increased serum total IgE and OVA-specific IgE levels in BALB/c mice.

#### Eosinophils

**Fig. 5:**
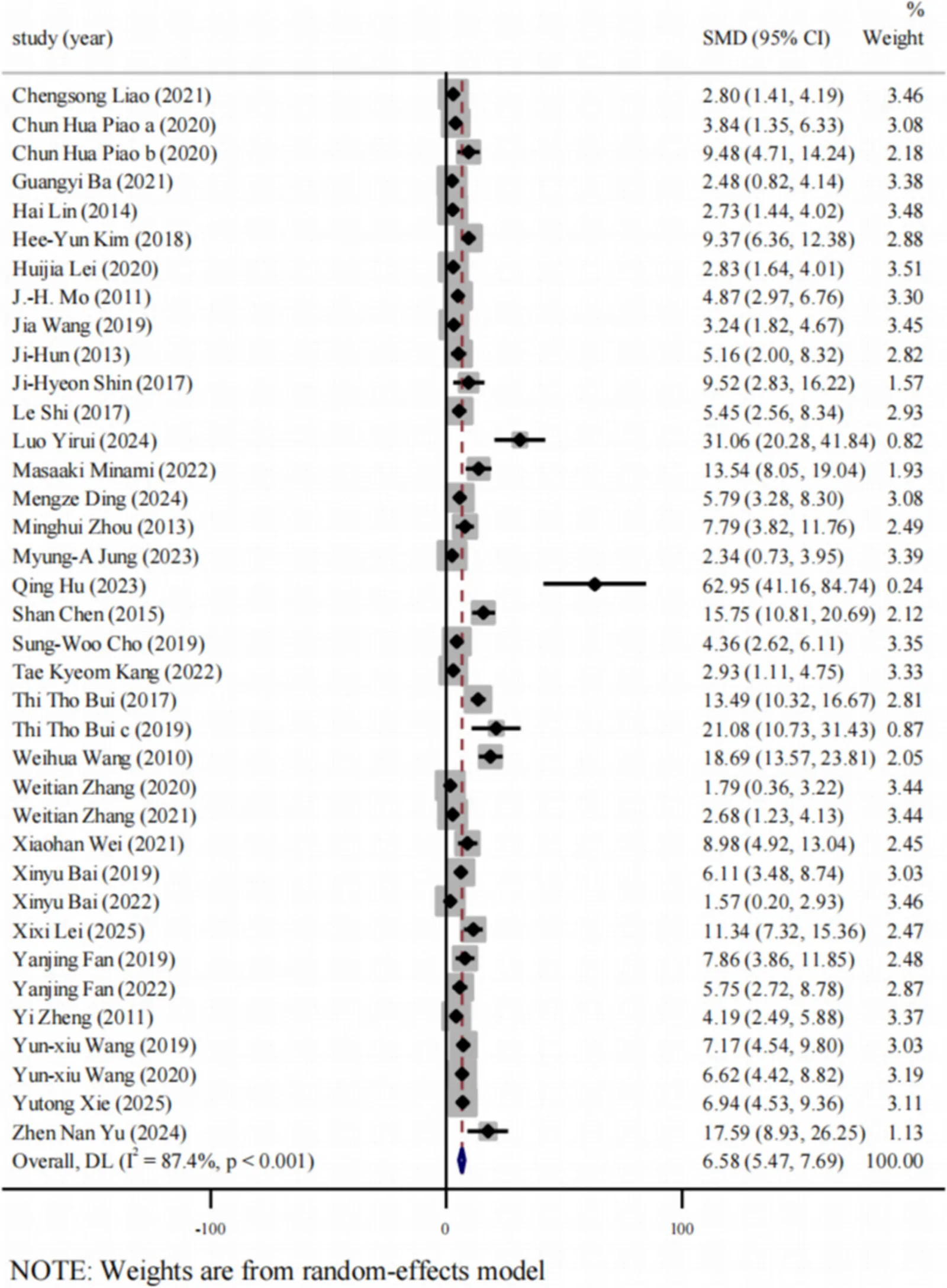
Meta-analysis results of eosinophil infiltration in the nasal mucosa of OVA/Al(OH)₃-induced AR model mice. (Note: Effect sizes are presented as SMDs with 95% CIs, and pooled analyses were performed using a random-effects model. Squares represent the effect sizes of individual studies, with the size of each square indicating the study weight; horizontal lines represent 95% CIs; and diamonds represent the pooled effect sizes)

The random-effects meta-analysis showed that, compared with the normal control group, the OVA combined with Al(OH)₃ model group exhibited a significant increase in the number of eosinophils in the nasal mucosa (SMD = 6.58, 95% CI: 5.47–7.69, *P* < 0.001, I² = 87.4%), as shown in Fig. 5. These findings suggest that this modeling method can significantly induce eosinophil infiltration in the nasal mucosa.

#### Meta-analysis of Th2-related cytokines in serum and nasal lavage fluid

**Fig. 6:**
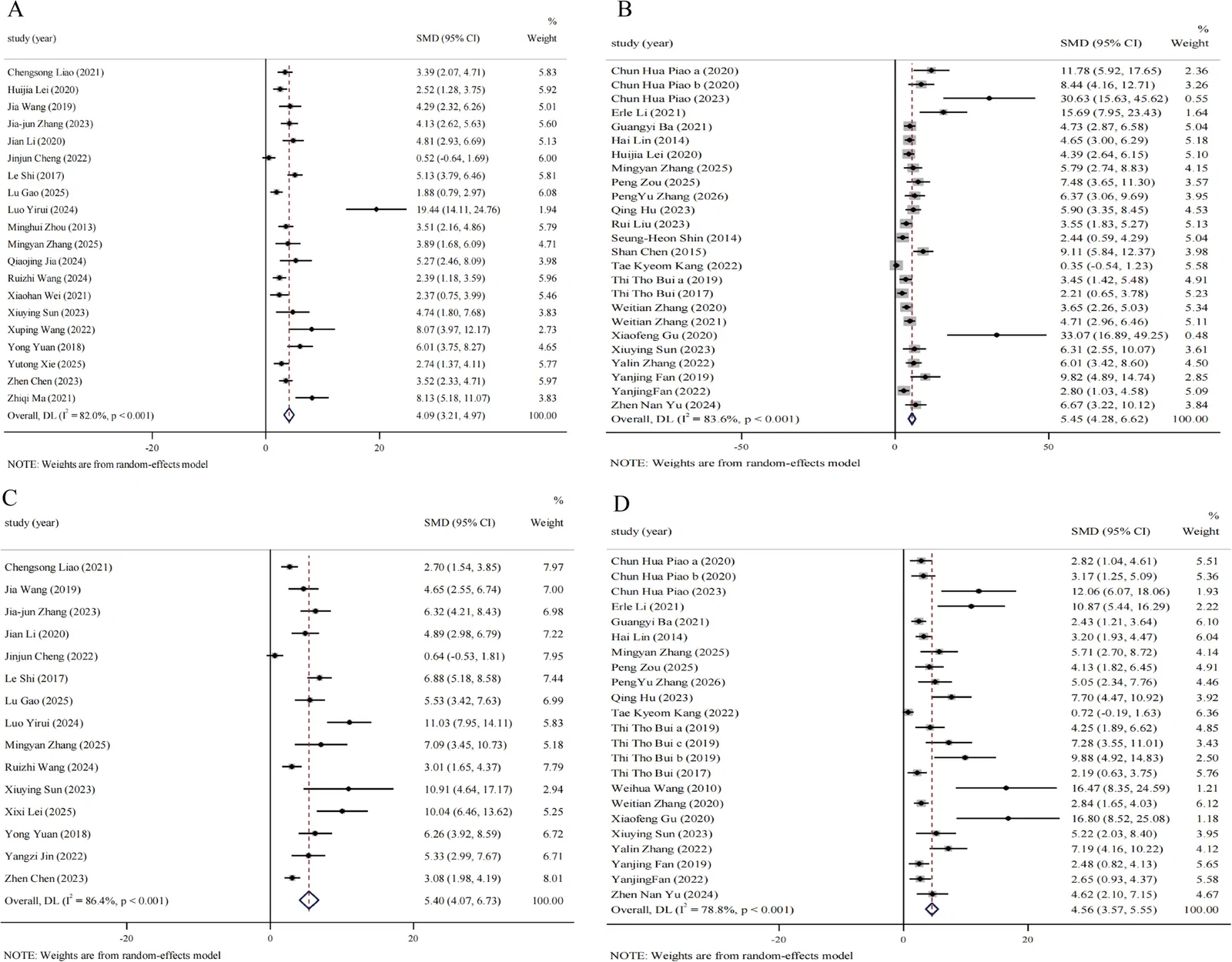
Meta-analysis results of Th2-related cytokines in OVA/Al(OH)₃-induced AR model mice (**A**), serum IL-4 level; **B**, IL-4 level in NLF; **C**, serum IL-5 level; **D**, IL-5 level in NLF. (Note: Effect sizes are presented as SMDs with 95% CIs, and pooled analyses were performed using a random-effects model. Squares represent the effect sizes of individual studies, with the size of each square indicating the study weight; horizontal lines represent 95% CIs; and diamonds represent the pooled effect sizes)

To further evaluate the Th2-related immunoinflammatory characteristics of the BALB/c mouse AR model induced by OVA combined with Al(OH)₃, random-effects meta-analyses were performed separately for IL-4 and IL-5 levels in serum and nasal lavage fluid (NLF). The results showed that, compared with the normal group, the model group had significantly increased serum IL-4 levels, with a pooled effect size of SMD = 4.09 (95% CI: 3.21–4.97, I² = 82.0%). IL-4 levels in NLF were also significantly elevated, with a pooled effect size of SMD = 5.45 (95% CI: 4.28–6.62, I² = 83.6%). Similarly, serum IL-5 levels were significantly increased in the model group, with a pooled effect size of SMD = 5.40 (95% CI: 4.07–6.73, I² = 86.4%). IL-5 levels in NLF were also markedly elevated, with a pooled effect size of SMD = 4.56 (95% CI: 3.57–5.55, I² = 78.8%), as shown in Fig. 6. These findings suggest that OVA combined with Al(OH)₃ modeling significantly increases IL-4 and IL-5 levels in both serum and the nasal local environment of BALB/c mice.

### Subgroup analysis and meta-regression of modeling parameters

To further investigate the potential influence of differences in modeling parameters on the effect sizes of BALB/c mouse models of allergic rhinitis induced by OVA combined with Al(OH)₃, subgroup analyses and meta-regression analyses were performed based on key doses used during model establishment. Because several potential moderating factors were insufficiently reported in the included studies, particularly information on the sex, age, and body weight of the experimental animals, the source and supplier of OVA, the endotoxin content of OVA, sample collection time points, and detection platforms, the completeness of data for these variables was inadequate and inconsistent across studies. Therefore, these factors were not included in the formal subgroup analyses or meta-regression models.

#### Subgroup analysis of parameters during the sensitization phase

To investigate the potential influence of OVA and Al(OH)₃ dose combinations during the sensitization phase on the effect sizes of the AR model, subgroup analyses were performed according to different OVA sensitization protocols, and their effects on behavioral, immunological, and inflammation-related outcome measures were evaluated separately. Overall, all sensitization protocols included in the analysis showed positive pooled SMDs, indicating that, compared with the normal control group, different OVA and Al(OH)₃ sensitization dose combinations could induce increases in AR-related outcome measures in the model group. However, subgroup differences were not entirely consistent across different outcome measures, suggesting that the effects of sensitization dose combinations on model phenotypes may be outcome-specific.

Regarding behavioral outcomes, the between-subgroup difference in the number of sneezing episodes within 10 min was statistically significant (*P* = 0.003), suggesting that different OVA and Al(OH)₃ dose combinations may have different effects on the intensity of the sneezing response. Among them, the 100 µg OVA plus 2 mg Al(OH)₃ subgroup showed the highest pooled effect size (SMD = 7.63, 95% CI: 3.68–11.58), followed by the 25 µg OVA plus 2 mg Al(OH)₃ subgroup (SMD = 7.42, 95% CI: 4.07–10.78). However, the between-subgroup differences were not statistically significant for the number of sneezing episodes within 15 min (*P* = 0.065), the number of nasal scratching events within 10 min (*P* = 0.242), or the number of nasal scratching events within 15 min (*P* = 0.766), indicating that differences among sensitization dose combinations were not consistent across other behavioral observation windows or behavioral indicators.

Regarding immunological and inflammation-related outcomes, the between-subgroup differences were not statistically significant for OVA-specific IgE (*P* = 0.407) or IL-4 levels in nasal lavage fluid (*P* = 0.126), suggesting that different sensitization dose combinations did not show clear differential effects on these indicators. In contrast, the between-subgroup differences were statistically significant for eosinophil infiltration in the nasal mucosa (*P* < 0.001) and IL-5 levels in nasal lavage fluid (*P* = 0.012). Among these, the 50 µg OVA plus 1 mg Al(OH)₃ regimen showed the highest pooled effect size for eosinophil infiltration (SMD = 9.32, 95% CI: 6.22–12.43), whereas the 25 µg OVA plus 2 mg Al(OH)₃ regimen showed the highest pooled effect size for IL-5 levels in nasal lavage fluid (SMD = 7.75, 95% CI: 3.03–12.46). In addition, the 100 µg OVA plus 2 mg Al(OH)₃ regimen showed a relatively large pooled effect size for OVA-specific IgE (SMD = 15.34, 95% CI: 6.22–24.45); however, the between-subgroup difference for this outcome was not statistically significant, and heterogeneity was high. To improve readability, detailed subgroup analyses according to OVA and Al(OH)_3_ sensitization dose combinations are provided in Supplementary Table S4.

#### Subgroup analysis of parameters during the challenge phase

Different intranasal OVA challenge protocols induced increases in AR-related behavioral, immunological, and local inflammatory indicators, with positive pooled SMDs observed across all subgroups. For behavioral indicators, the between-subgroup differences in the number of sneezing episodes within 15 min and the number of nasal scratching events within 15 min were not statistically significant across different intranasal challenge protocols (*P* = 0.288 and *P* = 0.971, respectively). Among them, the protocol involving seven consecutive intranasal challenges from days 0 to 6 with 400 µg OVA showed a relatively high pooled effect size for the number of sneezing episodes within 15 min (SMD = 6.33, 95% CI: 2.81–9.84), but the difference did not reach statistical significance compared with the 100 µg OVA protocol.

For immunological indicators, the between-subgroup differences in OVA-specific IgE were not statistically significant (*P* = 0.067), although the pooled effect sizes were relatively high across different challenge protocols. Among these, the protocol involving seven consecutive intranasal challenges from days 0 to 6 with 100 µg OVA showed the highest pooled effect size (SMD = 17.26, 95% CI: 8.07–26.45).

For local inflammatory indicators, eosinophil infiltration in the nasal mucosa differed significantly across different intranasal OVA challenge protocols (*P* = 0.030). Among these, the protocol involving seven consecutive intranasal challenges from days 0 to 6 with 400 µg OVA showed the highest pooled effect size (SMD = 8.26, 95% CI: 3.94–12.57), followed by the protocol involving five consecutive challenges from days 0 to 4 with 400 µg OVA (SMD = 8.15, 95% CI: 2.81–13.49). The between-subgroup difference in IL-4 levels in NLF was also statistically significant (*P* = 0.007), with the protocol involving five consecutive challenges from days 0 to 4 with 400 µg OVA showing the highest pooled effect size (SMD = 9.95, 95% CI: 1.32–18.59). In contrast, the between-subgroup difference in IL-5 levels in NLF was not statistically significant (*P* = 0.509). To improve readability, the subgroup analysis of core outcomes across different intranasal OVA challenge protocols is provided in Supplementary Table S5.

#### Meta-regression

To further explore potential sources of between-study heterogeneity, meta-regression analyses were performed for the main behavioral, immunological, and inflammation-related outcome measures using OVA and Al(OH)₃ sensitization dose combinations and intranasal OVA challenge protocols as moderator variables.

When the OVA/Al(OH)₃ sensitization protocol or dose regimen was used as the moderator variable, the meta-regression results for the main outcome measures did not reach statistical significance (all *P* > 0.05). However, 10-min sneezing frequency (*P* = 0.0798, adjusted R² = 32.34%) and nasal mucosal eosinophil infiltration (*P* = 0.1008, adjusted R² = 36.08%) showed certain trends, suggesting that differences in sensitization protocols may partially explain the heterogeneity in some behavioral and local inflammatory outcomes.

When the intranasal OVA challenge protocol was used as the moderator variable, the overall moderator effects for 15-min sneezing frequency, 15-min nasal rubbing frequency, OVA-specific IgE, nasal mucosal eosinophil infiltration, and IL-4 and IL-5 levels in nasal lavage fluid did not reach statistical significance (all *P* > 0.05). Among these outcomes, the adjusted R² values for nasal mucosal eosinophil infiltration and IL-4 levels in nasal lavage fluid were 10.51% and 14.62%, respectively, suggesting that the intranasal challenge protocol may explain a small proportion of the heterogeneity in local inflammation-related outcomes. The meta-regression analysis of the effects of modeling parameters on the effect sizes of core outcomes is presented in Table 4.

**Table 4:** Meta-regression analysis of the effects of modeling parameters on the effect sizes of core outcomes

Outcome	Moderator	k	*P* for moderator	Adjusted *R*² (%)	Residual I²(%)
15 min sneezing frequency	OVA/Al(OH)₃ sensitization protocol	14	0.2889	21.75	67.45
15 min rubbing frequency	OVA/Al(OH)₃ sensitization protocol	14	0.8740	-58.49	69.12
10 min sneezing frequency	OVA/Al(OH)₃ sensitization protocol	14	0.0798	32.34	83.45
10 min rubbing frequency	OVA/Al(OH)₃ sensitization protocol	14	0.4718	-14.47	80.03
OVA-specific IgE	OVA/Al(OH)₃ sensitization protocol	23	0.3672	-4.58	92.00
Nasal mucosal eosinophil infiltration	OVA/Al(OH)₃ sensitization protocol	22	0.1008	36.08	80.33
IL-4 in NLF	OVA/Al(OH)₃ sensitization protocol	19	0.7346	-52.10	73.59
IL-5 in NLF	OVA/Al(OH)₃ sensitization protocol	19	0.8130	-17.48	68.10
15 min sneezing frequency	Intranasal OVA challenge protocol	10	0.6590	-14.19	87.76
15 min rubbing frequency	Intranasal OVA challenge protocol	10	0.9060	-19.93	87.02
OVA-specific IgE	Intranasal OVA challenge protocol	11	0.5561	-19.22	86.95
Nasal mucosal eosinophil infiltration	Intranasal OVA challenge protocol	18	0.2967	10.51	72.36
IL-4 in NLF	Intranasal OVA challenge protocol	10	0.4986	14.62	71.87
IL-5 in NLF	Intranasal OVA challenge protocol	10	0.8693	—	51.56

k indicates the number of included studies or effect sizes in each meta-regression model. Adjusted R², proportion of between-study heterogeneity explained by the moderator after adjustment; Residual I², residual heterogeneity after inclusion of the moderator. Negative adjusted R² values indicate that the moderator did not improve model fit and should be interpreted as no explanatory contribution. “—” indicates that the value was not estimable or not applicable

Overall, the meta-regression analyses did not identify statistically significant moderator effects of the OVA/Al(OH)₃ sensitization protocol or intranasal OVA challenge protocol on the effect sizes of individual outcomes. In addition, the residual I² values remained moderate to high for most outcomes after inclusion of these moderators, indicating that a substantial proportion of between-study heterogeneity remained unexplained. These findings suggest that heterogeneity in BALB/c mouse models of allergic rhinitis induced by OVA/Al(OH)₃ may not be determined by a single modeling parameter, but rather by the combined influence of multiple experimental and methodological factors, including OVA and Al(OH)₃ doses, the number of sensitizations and challenges, experimental duration, sampling time points, detection methods, sample size, OVA source, endotoxin content, adjuvant formulation differences, and animal sex, age, and body weight.Because most potential moderators were insufficiently reported in the original studies, the explanatory power of the meta-regression models was limited. Therefore, the meta-regression results in this study should be interpreted as exploratory analyses that may help identify potential sources of heterogeneity, rather than as confirmatory evidence that specific modeling parameters independently affect outcome effect sizes.

#### Sensitivity analysis and assessment of publication bias

##### Sensitivity analysis

**Fig. 7:**
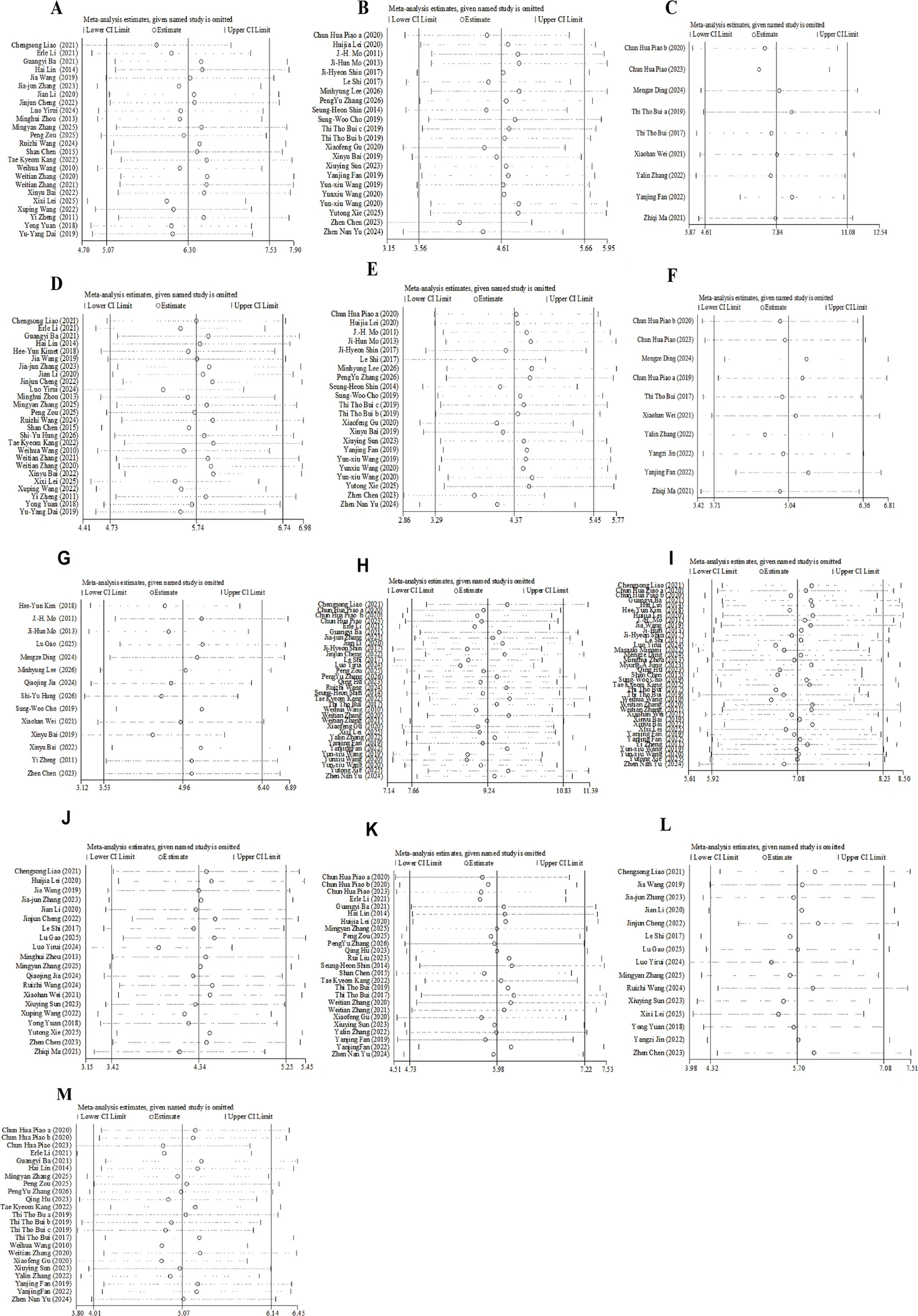
Leave-one-out sensitivity analysis results for each outcome measure. **A**, number of sneezing episodes within 10 min; **B**, number of sneezing episodes within 15 min; **C**, number of sneezing episodes within 20 min; **D**, number of nasal scratching/rubbing events within 10 min; **E**, number of nasal scratching/rubbing events within 15 min; **F**, number of nasal scratching/rubbing events within 20 min; **G**, serum total IgE; **H**, OVA-specific IgE; **I**, eosinophil infiltration in the nasal mucosa; **J**, serum IL-4; **K**, IL-4 in NLF **L**, serum IL-5; and **M**, IL-5 in NLF. Note: Each row represents the pooled SMD recalculated after excluding the corresponding study. The circle indicates the recalculated pooled effect estimate, and the horizontal line represents its 95% CI. The solid vertical line indicates the overall pooled SMD based on all studies, whereas the dashed vertical lines indicate the corresponding 95% CI

Sensitivity analyses of the main outcome measures were performed using the leave-one-out method. The results showed that, after sequential exclusion of individual studies, the direction of the pooled effects for all main outcomes remained unchanged, suggesting that the overall findings were relatively stable. Among these outcomes, the effect sizes and confidence intervals for eosinophil infiltration and IL-5 levels in nasal lavage fluid showed some fluctuation, which may be related to the high between-study heterogeneity. Overall, no single study was found to have a decisive influence on the pooled results, indicating that the main meta-analysis findings of this study had a certain degree of robustness, as shown in Fig. 7.

##### Assessment of publication bias

**Fig. 8:**
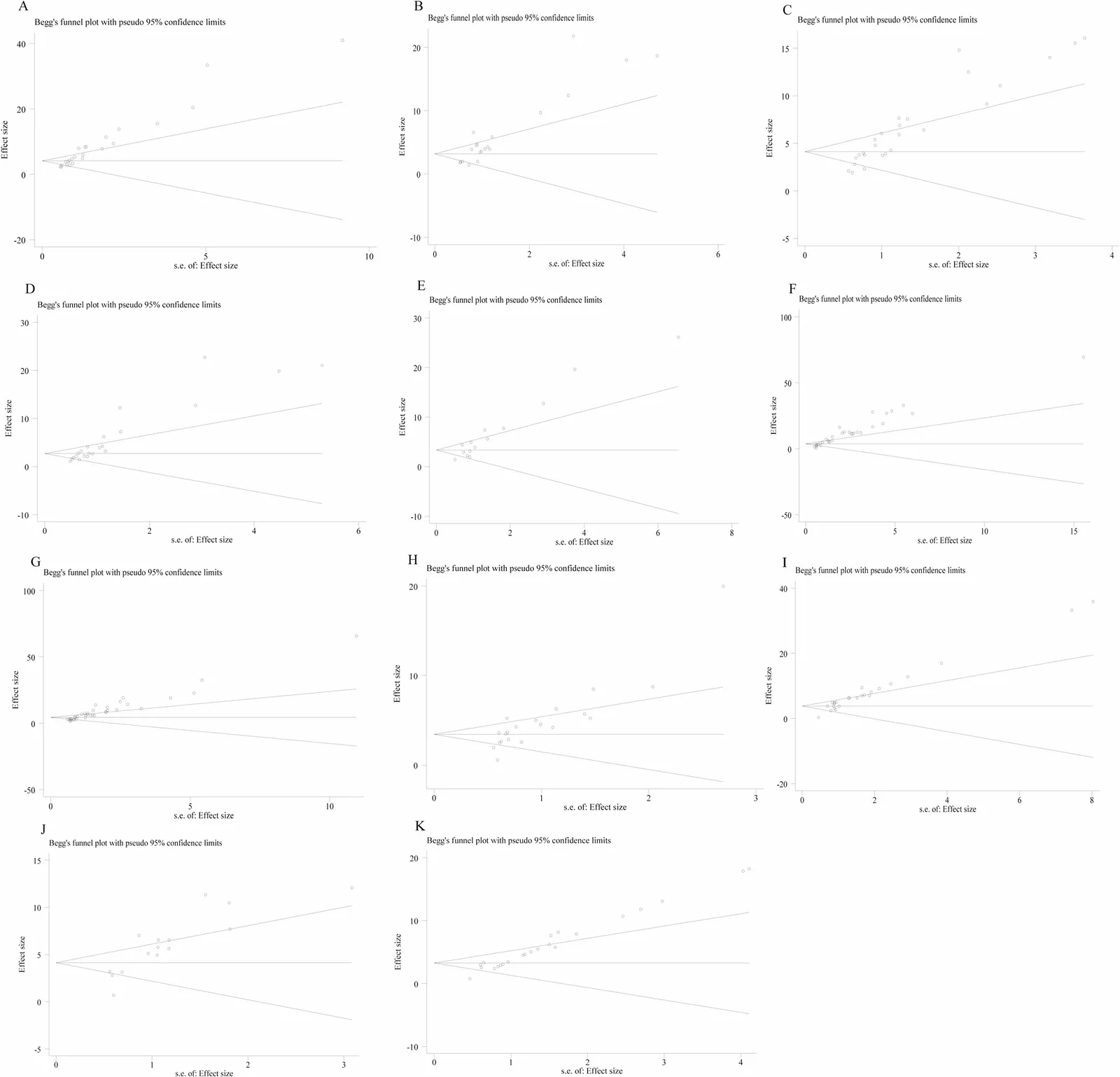
Funnel plots for the assessment of publication bias in the main outcome measures. **A**, number of sneezing episodes within 10 min; **B**, number of sneezing episodes within 15 min; **C**, number of nasal scratching/rubbing events within 10 min; **D**, number of nasal scratching/rubbing events within 15 min; **E**, serum total IgE; **F**, OVA-specific IgE; **G**, eosinophil infiltration in the nasal mucosa; **H**, serum IL-4; **I**, IL-4 in NLF; **J**, serum IL-5; and **K**, IL-5 in NLF. Publication bias for the main outcome measures was assessed using Begg’s rank correlation test

Publication bias for the main outcome measures was assessed using Begg’s rank correlation test. The results showed that, among the behavioral outcomes, the numbers of sneezing episodes within 10 min and 15 min, as well as the numbers of nasal scratching or rubbing events within 10 min and 15 min, were statistically significant (all Pr > |z| < 0.001). Among the immunological and inflammation-related outcomes, serum total IgE (Pr > |z| = 0.003), OVA-specific IgE (Pr > |z| < 0.001), eosinophil infiltration in the nasal mucosa (Pr > |z| < 0.001), serum IL-4 (Pr > |z| < 0.001), IL-4 in nasal lavage fluid (Pr > |z| < 0.001), serum IL-5 (Pr > |z| = 0.008), and IL-5 in nasal lavage fluid (Pr > |z| < 0.001) were also statistically significant, as shown in Fig. 8. These findings suggest that the main outcome measures in this study may be subject to publication bias or small-study effects. Considering that most of the included studies were small-sample animal experiments and that differences existed in modeling protocols, intervention doses, detection time points, and outcome reporting methods, the pooled effect sizes should be interpreted comprehensively in conjunction with the heterogeneity analysis, sensitivity analysis, and trim-and-fill results.

To further assess the influence of publication bias on the pooled effect sizes, the trim-and-fill method was applied. Because heterogeneity tests were statistically significant for all outcome measures (Q test, all *P* < 0.001), the results were primarily interpreted based on the random-effects model. After trim-and-fill adjustment, the pooled effect size for the number of sneezing episodes within 10 min was 4.572 (95% CI: 3.281–5.863, *P* < 0.001, Q = 237.392), and that for the number of sneezing episodes within 15 min was 4.244 (95% CI: 3.237–5.251, *P* < 0.001, Q = 130.847). The pooled effect size for the number of nasal scratching or rubbing events within 10 min was 4.194 (95% CI: 3.157–5.230, *P* < 0.001, Q = 201.358), and that for the number of nasal scratching or rubbing events within 15 min was 4.032 (95% CI: 2.996–5.067, *P* < 0.001, Q = 156.592). No trimming and filling was performed for the number of nasal scratching or rubbing events within 15 min, indicating that the results remained unchanged before and after adjustment.

**Fig. 9:**
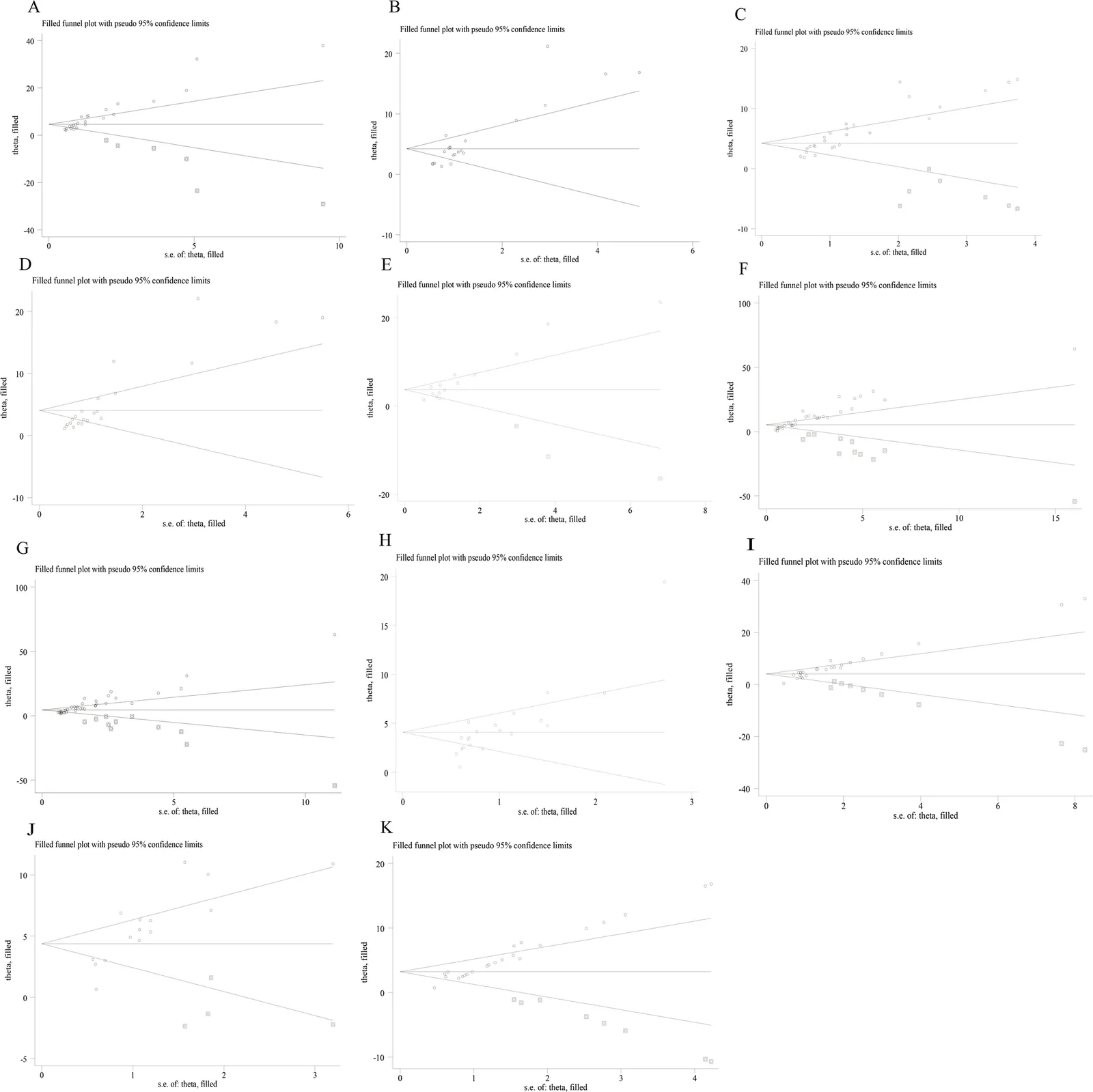
Trim-and-fill analysis results for publication bias in the main outcome measures. **A**, number of sneezing episodes within 10 min; **B**, number of sneezing episodes within 15 min; **C**, number of nasal scratching/rubbing events within 10 min; **D**, number of nasal scratching/rubbing events within 15 min; **E**, serum total IgE; **F**, OVA-specific IgE; **G**, eosinophil infiltration in the nasal mucosa; **H**, serum IL-4; **I**, IL-4 in NLF; **J**, serum IL-5; and **K**, IL-5 in NLF

Among immunological and inflammation-related outcomes, the pooled effect size for serum total IgE was 3.691 (95% CI: 2.198–5.183, *P* < 0.001, Q = 101.479), that for OVA-specific IgE was 5.331 (95% CI: 3.775–6.888, *P* < 0.001, Q = 518.954), and that for eosinophil infiltration in the nasal mucosa was 4.586 (95% CI: 3.372–5.800, *P* < 0.001, Q = 455.174). The pooled effect size for serum IL-4 was 4.089 (95% CI: 3.209–4.968, *P* < 0.001, Q = 105.556), that for IL-4 in nasal lavage fluid was 4.042 (95% CI: 2.883–5.202, *P* < 0.001, Q = 202.458), that for serum IL-5 was 4.380 (95% CI: 3.075–5.685, *P* < 0.001, Q = 131.571), and that for IL-5 in nasal lavage fluid was 3.217 (95% CI: 2.170–4.263, *P* < 0.001, Q = 164.574), as shown in Fig. 9. These results showed that all adjusted effect sizes remained statistically significant (*P* < 0.001). This finding suggests that, although significant publication bias was present and may have led to overestimation of the effect sizes, the core conclusion that OVA combined with Al(OH)₃ can effectively induce an AR model remains robust.

### GRADE assessment of evidence quality

The certainty of evidence for the major outcomes was assessed using an adapted GRADE approach for preclinical animal studies. As shown in Table 5, the certainty of evidence was rated as very low for most major outcomes. Although the pooled effect estimates were statistically significant and generally supported that the OVA combined with aluminum hydroxide protocol induced AR-related behavioral, immunological, and histopathological phenotypes, the certainty of evidence was limited by several methodological and statistical concerns.

**Table 5:** GRADE assessment of the certainty of evidence

Outcome	No. of studies	Effect size, SMD (95% CI)	Heterogeneity, I²	Downgrading domains	Certainty of evidence
10 min Sneezing frequency	24	5.85 (4.68–7.02)	85.7%	Risk of bias; inconsistency; publication bias	Very low
15 min Sneezing frequency	22	4.24 (3.24–5.25)	84.0%	Risk of bias; inconsistency; publication bias	Very low
20 min Sneezing frequency	9	7.21 (4.17–10.26)	89.3%	Risk of bias; inconsistency; publication bias; imprecision	Very low
10 min Nasal rubbing/scratching	26	5.33 (4.38–6.29)	81.5%	Risk of bias; inconsistency; publication bias	Very low
15 min Nasal rubbing/scratching	22	4.03 (3.00–5.07)	86.6%	Risk of bias; inconsistency; publication bias	Very low
20 min Nasal rubbing/scratching	10	4.66 (3.39–5.92)	65.7%	Risk of bias; inconsistency; publication bias; imprecision	Very low
OVA-specific IgE	33	8.53 (7.01–10.05)	91.5%	Risk of bias; inconsistency; publication bias	Very low
Serum IgE	14	4.51 (3.16–5.86)	82.0%	Risk of bias; inconsistency; publication bias	Very low
Nasal mucosal eosinophil infiltration	37	6.58 (5.47–7.69)	87.4%	Risk of bias; inconsistency; publication bias	Very low
Serum IL-4	20	4.09 (3.21–4.97)	82.0%	Risk of bias; inconsistency; publication bias	Very low
NLF IL-4	25	5.45 (4.28–6.62)	83.6%	Risk of bias; inconsistency; publication bias	Very low
Serum IL-5	15	5.40 (4.07–6.73)	86.4%	Risk of bias; inconsistency; publication bias	Very low
NLF IL-5	22	4.56 (3.57–5.55)	78.8%	Risk of bias; inconsistency; publication bias	Very low

The initial certainty of evidence was rated as low because all evidence was derived from preclinical animal studies. For each outcome, the five GRADE downgrading domains—risk of bias, inconsistency, indirectness, imprecision, and publication bias—were assessed; only downgraded domains are shown. Indirectness was generally not downgraded at the preclinical population/animal model, intervention, comparator, and outcome (PICO) level because the included studies used OVA/Al(OH)₃-induced mouse models of allergic rhinitis (AR), and the main comparisons and outcomes were aligned with the predefined preclinical question. Outcomes, including AR-related behavioral symptom scores, inflammatory/immune markers, and histopathological changes, were considered relevant to this model. Inconsistency was assessed using I²; imprecision was judged by limited studies/comparisons, small sample sizes, or wide CIs; and publication bias was assessed by funnel plot asymmetry and/or Begg’s test, where applicable. With no upgrading applied and downgrading present in at least one domain, the final certainty was rated as very low

The main reasons for downgrading were risk of bias due to insufficient reporting of methodological details in the included animal studies, inconsistency caused by moderate to substantial between-study heterogeneity, imprecision for outcomes supported by a limited number of studies or comparisons and/or wide confidence intervals, and publication bias suggested by funnel plot asymmetry or Begg’s test. Therefore, while the findings support the use of OVA combined with aluminum hydroxide as a reference protocol for inducing AR-related phenotypes in mouse models, the overall strength of evidence remains limited. These results should be interpreted cautiously and regarded as supportive rather than definitive evidence for recommending reference modeling protocols.

## Discussion

### Core findings of the meta-analysis

The results of this study showed that OVA combined with Al(OH)₃ effectively induced AR-related phenotypes in BALB/c mice, as demonstrated by increased behavioral symptoms such as sneezing and nasal scratching, accompanied by elevated OVA-specific IgE levels, enhanced expression of Th2-type inflammatory cytokines, and eosinophil infiltration in the nasal mucosa. Overall, the evidence from multiple outcome measures suggests that three intraperitoneal sensitizations followed by intranasal OVA challenges for either 5 or 7 consecutive days may produce relatively favorable model-induction effects.

It is noteworthy that the modeling protocols most frequently used in the literature did not necessarily show the highest effect sizes across all outcome measures. The effects of different OVA/Al(OH)₃ dose combinations on model induction appeared to be outcome-specific. Specifically, 100 µg OVA plus 2 mg Al(OH)₃ showed relatively large effect sizes for behavioral symptoms, whereas 50 µg OVA plus 1 mg Al(OH)₃ more prominently reflected eosinophil infiltration in the nasal mucosa, and 25 µg OVA plus 2 mg Al(OH)₃ showed a relatively large effect size for IL-5 levels in NLF. These findings suggest that different modeling protocols may differentially affect the behavioral phenotype, systemic antigen-specific immune response, and local inflammatory response of AR models. Therefore, researchers should select the most appropriate modeling parameters according to their primary observational endpoints.

### Immunological interpretation of the OVA combined with Al(OH)₃ model

The allergic rhinitis model induced by OVA combined with Al(OH)₃ is a classical Th2-skewed model of allergic inflammation. As a commonly used model antigen, OVA, when used for sensitization in combination with the Al(OH)₃ adjuvant, can enhance the host’s OVA-specific immune response [24].During the sensitization phase, OVA is taken up, processed, and presented by antigen-presenting cells with the assistance of the Al(OH)₃ adjuvant, thereby promoting the differentiation of naïve T cells into Th2 cells and inducing IgE class switching in B cells, resulting in the production of total IgE and OVA-specific IgE [25].During the intranasal challenge phase, re-exposure of the nasal mucosa to OVA can lead to cross-linking of OVA-specific IgE bound to high-affinity FcεRI receptors on the surface of mast cells and basophils, thereby inducing the release of inflammatory mediators such as histamine and leukotrienes. This process subsequently triggers immediate allergic responses, including sneezing, nasal itching, and nasal scratching [26]. Meanwhile, Th2-type cytokines further amplify the local inflammatory response. Among them, IL-4 is primarily involved in maintaining the Th2 response and promoting IgE production, whereas IL-5 is closely associated with the recruitment, activation, and survival of eosinophils [27]. Therefore, the indicators included in this study, including IgE, OVA-specific IgE, IL-4, IL-5, and eosinophil infiltration in the nasal mucosa, can reflect the modeling efficacy of the OVA combined with Al(OH)₃ model from the perspectives of systemic immune activation, Th2-type inflammatory responses, and local histopathological changes. It should be noted that the OVA/Al(OH)₃ model is essentially an experimental model driven by an artificial antigen and adjuvant. Although it has good reproducibility and prominent Th2 immune characteristics, it is not fully equivalent to allergic rhinitis in humans caused by natural exposure to allergens such as house dust mites or pollen. Therefore, this model is more suitable for studies of immunological mechanisms, pharmacodynamic evaluation, and comparisons of modeling parameters.

### Modeling parameters, animal strain, and sources of heterogeneity

This study further showed that sensitization dose, adjuvant dose, intranasal challenge dose, number of challenges, and challenge duration may all influence model effect sizes and represent important sources of between-study heterogeneity. During the sensitization phase, significant subgroup differences were observed among different OVA and Al(OH)₃ dose combinations in the number of sneezing episodes within 10 min, eosinophil infiltration in the nasal mucosa, and IL-5 levels in NLF, suggesting that the sensitization dose and adjuvant dose may affect the intensity of behavioral symptoms and local Th2-type inflammatory responses.

Among these protocols, 100 µg OVA plus 2 mg Al(OH)₃ showed a relatively pronounced effect on inducing short-term sneezing responses, 50 µg OVA plus 1 mg Al(OH)₃ showed a more prominent effect on inducing eosinophil infiltration in the nasal mucosa, and 25 µg OVA plus 2 mg Al(OH)₃ showed a relatively large effect size for IL-5 levels in NLF. In contrast, subgroup differences were not significant for the number of sneezing episodes within 15 min, the number of nasal scratching events, OVA-specific IgE, or IL-4 levels in NLF, indicating that the influence of sensitization dose combinations was dependent on the specific outcome measure.

During the intranasal challenge phase, different OVA challenge protocols mainly affected local inflammatory indicators. Overall, continuous challenge with 400 µg OVA for 5–7 days showed relatively favorable effects. Specifically, the protocol involving seven consecutive challenges from days 0 to 6 with 400 µg OVA showed the highest effect size for eosinophil infiltration in the nasal mucosa, whereas the protocol involving five consecutive challenges from days 0 to 4 with 400 µg OVA showed the highest effect size for IL-4 levels in NLF. In contrast, no significant differences were observed among different challenge protocols in the number of sneezing episodes within 15 min, the number of nasal scratching events within 15 min, OVA-specific IgE, or IL-5 levels in NLF, suggesting that the effects of intranasal challenge parameters on behavioral symptoms and systemic immunological indicators were not consistent.

Differences in animal strain may also represent a potential source of heterogeneity. Compared with C57BL/6 mice, BALB/c mice exhibit more pronounced Th2-type immune responses, including higher serum IgE levels, increased IL-4 and IL-5 expression, and stronger local inflammatory responses [28]. Therefore, BALB/c mice are often considered more suitable for establishing allergic rhinitis models characterized predominantly by Th2-type inflammation. In contrast, C57BL/6 mice have a different immunological background, and their responses to allergen sensitization and intranasal challenge may differ, thereby affecting model phenotypes and the magnitude of effect sizes. Qidi Zhang et al. found that, under the same dosing regimen, BALB/c and C57BL/6 mice exhibited significant differences in immune responses. BALB/c mice showed mild local allergic inflammatory responses but strong systemic immune responses, whereas C57BL/6 mice exhibited stronger local allergic inflammatory responses and relatively moderate systemic immune responses [29].

Compared with BALB/c mice, strains such as C57BL/6, ICR, 129/Sv, and Kunming mice differ in their immunological response tendencies or genetic backgrounds, which may lead to inconsistencies in the intensity of inflammatory responses, phenotypic stability, and magnitude of effect sizes in AR models. The quantitative analyses in this study were primarily based on BALB/c mice, which helped reduce biological heterogeneity caused by differences in genetic background. However, this approach also limits the generalizability of the findings to other mouse strains. In the future, when sufficient data are available, animal strain should be further analyzed as a subgroup variable or moderator in meta-regression.

In addition, substantial publication bias, as described in Assessment of publication bias, may also have contributed to the observed high pooled effect sizes and may have masked between-study heterogeneity. Because studies with negative or null findings are less likely to be published, the available literature may systematically overestimate the true effect size of this model. Moreover, factors such as animal sex, age, body weight, detection time points, ELISA kits, tissue staining methods, regions selected for eosinophil counting, and insufficient reporting of randomization, blinding, and allocation concealment may also contribute to inconsistencies in outcome measurements. Therefore, the extremely high heterogeneity observed in this study, with I² values frequently exceeding 80%, likely reflects the combined effects of differences in modeling protocols, animal characteristics, detection methods, study quality, and publication bias.

### Scope of applicability and cautious interpretation of the findings

The modeling parameter recommendations provided in this study should be understood as reference guidance based on the current literature evidence in the context of BALB/c mice, which may be subject to substantial publication bias, rather than as an absolutely optimal protocol applicable to all experimental purposes and all outcome measures. Researchers should validate and adjust the recommended protocol according to their specific experimental objectives, primary observational endpoints, and laboratory conditions. Based on the integrated behavioral, immunological, and histopathological outcomes, three intraperitoneal sensitizations, such as on days 0, 7, and 14, combined with daily intranasal OVA challenges for either 5 or 7 consecutive days may serve as a practical basic framework for establishing the OVA/alum-induced allergic rhinitis model.

Within this framework, the OVA sensitization dose should be selected in a context-specific manner according to the primary research endpoint. For studies focusing on behavioral endpoints, such as sneezing, the 100 µg OVA plus 2 mg Al(OH)₃ regimen may be preferentially considered. For studies focusing on histopathological endpoints, such as eosinophil infiltration, the 50 µg OVA plus 1 mg Al(OH)₃ regimen appears to have a more pronounced advantage. The 25 µg OVA plus 2 mg Al(OH)₃ regimen showed a relatively large effect size in inducing local IL-5 expression. In addition, continuous challenge with 400 µg OVA for 5–7 days may serve as a preferred reference for the intranasal challenge dose, as it showed relatively large effect sizes for eosinophil infiltration in the nasal mucosa and IL-4 levels in NLF, suggesting that this regimen may be more favorable for inducing local Th2-type inflammatory responses in the nasal mucosa.

It should be noted that the required intensity of model induction may vary depending on the research objective. For drug screening or mechanistic validation studies, modeling protocols with more pronounced inflammatory phenotypes and larger effect sizes may be selected. In contrast, for intervention dose–response studies or experiments requiring a model of moderate severity, the intensity of sensitization or challenge should be appropriately reduced to avoid ceiling effects caused by excessive symptom saturation.Therefore, when applying the conclusions of this study, researchers should pay close attention to the following points. First, all of the above recommendations are strictly limited to the BALB/c strain and should not be directly extrapolated to other mouse strains, such as C57BL/6 or ICR mice. Second, substantial publication bias exists in this field (Begg’s test, *P* < 0.001), and the pooled effect sizes presented in the literature may be at risk of overestimation; therefore, caution is warranted when estimating the expected model intensity. Third, the protocol provided in this study should be regarded as a benchmark protocol for priority testing and reference, rather than as a fixed standard that must be followed. Researchers should flexibly adjust and validate the protocol according to their specific experimental objectives, expected model intensity, and pilot experimental results.

## Strengths and limitations

The main strength of this study is that it focused on the OVA combined with Al(OH)₃-induced animal model of allergic rhinitis itself, rather than on pharmacological interventions. By systematically comparing key modeling parameters, including the number of sensitizations, number of intranasal challenges, and OVA/Al(OH)₃ dose combinations, this review provides a reference for model standardization. In addition, behavioral, immunological, and histopathological outcomes were comprehensively evaluated, allowing a more integrated assessment of model induction efficacy. The analysis was mainly based on BALB/c mice, which reduced, to some extent, biological variability caused by different genetic backgrounds and improved the comparability of modeling parameters.

Several limitations should also be acknowledged. First, significant publication bias and moderate to substantial heterogeneity were observed for several major outcomes. Moreover, most comparisons were based on indirect evidence across different studies rather than direct comparisons of modeling protocols. Therefore, the identification of a potentially optimal protocol should be interpreted cautiously in relation to the outcome type, heterogeneity level, and number of included studies. Second, because the analysis was mainly based on BALB/c mice and predominantly involved intraperitoneal sensitization followed by intranasal challenge, the findings cannot be directly extrapolated to other mouse strains or administration routes. In addition, the OVA/Al(OH)₃ model is an artificial antigen- and adjuvant-driven model, which differs from natural allergen exposure in humans and therefore has limited translational generalizability. Third, incomplete reporting of experimental details, including OVA source, endotoxin levels, animal sex, age, randomization, blinding, sampling time points, and detection methods, may have introduced bias and limited further exploration of heterogeneity. The limited reporting of outcomes such as IgG1, IL-13, goblet cell hyperplasia, epithelial injury, and nasal mucosal barrier function also restricted a comprehensive evaluation of the model mechanisms and pathological characteristics.

Furthermore, an adapted GRADE approach was applied to assess the certainty of evidence for major behavioral, immunological, and histopathological outcomes. Overall, the certainty of evidence was low to very low, mainly due to methodological limitations, inconsistency, imprecision, and publication bias. Therefore, although the pooled results support the ability of OVA combined with Al(OH)₃ to induce AR-related phenotypes in mouse models, the identified modeling protocols should be regarded as supportive rather than definitive evidence for model standardization.

## Conclusion

Through a systematic review and meta-analysis, this study compared key modeling parameters of the BALB/c mouse model of allergic rhinitis induced by OVA combined with Al(OH)₃. The results showed that this model can effectively induce AR-related phenotypes.Considering the available evidence and the potential influence of publication bias, three intraperitoneal sensitizations followed by 5 or 7 consecutive days of intranasal OVA challenges may serve as a practical basic modeling protocol.

The sensitization dose should be selected according to the primary research endpoint. For example, 100 µg OVA plus 2 mg Al(OH)₃ may be preferred for studies focusing on behavioral outcomes, whereas 50 µg OVA plus 1 mg Al(OH)₃ may be preferred for studies focusing on eosinophil infiltration.This recommendation should be regarded as a prioritized reference for modeling protocols in BALB/c mice rather than a fixed standard applicable to all experimental conditions. Future standardized and prospective animal experiments are still needed to further validate the effects of different modeling parameters on model stability, inflammatory intensity, and translational relevance.

## Supplementary Information

Supplementary Material 1.

## Data Availability

All data analyzed in this study are obtained from previously published articles. The datasets used and analyzed during the present study are available from the corresponding author on reasonable request.

## Abbreviations

AR: Allergic rhinitis
Al(OH)₃: Aluminum hydroxide
IgE: Immunoglobulin E
OVA: Ovalbumin
IL4: Interleukin-4
IL5: Interleukin-5
IL13: Interleukin-13
Th2: T helper 2
95% CI: 95% confidence interval
SEM: Standard error of the mean
SD: Standard deviation
SMD: Standardized mean difference
NLF: Nasal lavage fluid
PICO: Population/animal model, Intervention, Comparator, and Outcome
