# Supplementary material for: Parameter analysis of an ovalbumin combined with aluminum hydroxide adjuvant-induced allergic rhinitis model in BALB/c mice: a systematic review and meta-analysis
Source: BMC Immunol. 2026 Jul 20;27:100. doi: 10.1186/s12865-026-00880-2 (PMC13711739; doi:10.1186/s12865-026-00880-2)
Supplement: Supplementary file 1 [file 12865_2026_880_MOESM1_ESM.docx]

1 PRISMA flow diagram

**Identification of studies via databases and registers**

**Identification**

Records identified from*:

Broad search without aluminum hydroxide terms:

PubMed (n = 750)

Embase (n = 1059)

Web of Science (n = 867)

Cochrane Library (n = 8)

Supplementary aluminum hydroxide-specific search:
PubMed (n = 51; additional unique records = 0)
Embase (n = 115; additional unique records = 0)
Web of Science (n = 66; additional unique records = 0)
Cochrane Library (n = 0; additional unique records = 0)
Records identified before deduplication (n = 2684)

Records removed *before screening*:

Duplicate records removed (n = 856)

Records excluded**

Reviews, conference abstracts, or other non-original records (n = 356)

Non-animal studies (n = 223)
Language restrictions (n = 65)
Retracted or withdrawn articles (n = 13)

**Screening**

Records screened

(n = 1828)

Reports not retrieved

No access to full text (n = 36)

Reports sought for retrieval

(n = 1171)

Reports excluded:

Data could not be extracted(n = 215)

Insufficient or incomplete modeling parameters (n = 193)

Fewer than two core model evaluation outcomes reported (n = 230)

Allergic rhinitis combined with other airway diseases (n = 397)

Non-mouse animal studies(n =25)

Reports assessed for eligibility

(n = 1135)

Studies not included in quantitative synthesis:
129/Sv mice (n = 1)
ICR mice (n = 2)
Kunming mice (n = 1)

C57BL/6 mice (n =7)

Studies included in review

(n = 75)

**Included**

Studies included in primary meta-analysis

BALB/c mice (n= 64)

C57BL/6 mice (n=7)

Sprague-Dawley rats (n=5)

**2 Supplementary Material 1: Search strategies1**

**Table 1.** Search strategy for PubMed database

| ID | Search | Hits |
| --- | --- | --- |
| #1 | "allergic rhinitis"[Title/Abstract] OR "rhinitis allergic"[Title/Abstract] | 24896 |
| #2 | "Mice"[Title/Abstract] OR "Mouse"[Title/Abstract] | 1653479 |
| #3 | "Ovalbumin"[Title/Abstract] OR "OVA"[Title/Abstract] | 33279 |
| #4 | #1 AND #2 AND #3 | 750 |
| Last Run Date: 2026-04-18 | | |

**Table 2.** Search strategy for EMBASE database

| ID | Search | Hits |
| --- | --- | --- |
| #1 | 'allergic rhinitis'/exp | 68278 |
| #2 | 'allergic rhinitis':ab,ti OR 'rhinitis, allergic':ab,ti OR 'atopic rhinitis':ab,ti OR 'eosinophilic rhinitis':ab,ti OR 'allergic rhinopathy':ab,ti | 38956 |
| #3 | #1 OR #2 | 72913 |
| #4 | 'ovalbumin'/exp | 32755 |
| #5 | 'ovalbumin':ab,ti OR 'ova':ab,ti OR 'ovalbumen':ab,ti | 43464 |
| #6 | #4 OR #5 | 52178 |
| #7 | 'mouse'/exp | 2607698 |
| #8 | 'mice':ab,ti OR 'mus':ab,ti OR 'newborn mice':ab,ti | 1670771 |
| #9 | #7 OR #8 | 2751715 |
| #10 | #3 AND #6 AND #9 | 1059 |
| **Last Run Date: 2026-04-18** | | |

**Table 3.** Search strategy for Web of Science database

| ID | Search | Hits |
| --- | --- | --- |
| #1 | Allergic Rhinitis (Topic) OR rhinitis, allergic (Topic) | 28349 |
| #2 | Mice (Topic) OR Mouse (Topic) OR Mus (Topic) | 1719533 |
| #3 | Ovalbumin (Topic) OR OVA (Topic) | 30338 |
| #4 | #1 AND #2 AND #3 | 867 |
| Last Run Date:2026-04-18 | | |

**Table 4.** Search strategy for Cochrane database

| ID | Search | Hits |
| --- | --- | --- |
| #1 | (Allergic Rhinitis):ti,ab,kw OR (rhinitis, allergic):ti,ab,kw (Word variations have been searched) | 9576 |
| #2 | (Mus):ti,ab,kw OR (Mouse):ti,ab,kw OR (Mice):ti,ab,kw | 7196 |
| #3 | (Ovalbumin):ti,ab,kw OR (OVA):ti,ab,kw | 1116 |
| #4 | #1 and #2 and #3 | 8 |
| Last Run Date:2026-04-18 | | |

**Search strategies 2**

**Table 5.** Search strategy for PubMed database

| ID | Search | Hits |
| --- | --- | --- |
| #1 | "allergic rhinitis"[Title/Abstract] OR "rhinitis allergic"[Title/Abstract] | 24,896 |
| #2 | "Mice"[Title/Abstract] OR "Mouse"[Title/Abstract] | 1,653,479 |
| #3 | "Ovalbumin"[Title/Abstract] OR "OVA"[Title/Abstract] | 33,279 |
| #4 | "Alum"[Title/Abstract] OR "aluminum hydroxide"[Title/Abstract] | 8,285 |
| #5 | #1 AND #2 AND #3 AND #4 | 51 |
| Last Run Date: 2026-04-18 | | |

**Table 6.** Search strategy for EMBASE database

| ID | Search | Hits |
| --- | --- | --- |
| #1 | 'allergic rhinitis'/exp | 68278 |
| #2 | 'allergic rhinitis':ab,ti OR 'rhinitis, allergic':ab,ti OR 'atopic rhinitis':ab,ti OR 'eosinophilic rhinitis':ab,ti OR 'allergic rhinopathy':ab,ti | 38956 |
| #3 | #1 OR #2 | 72913 |
| #4 | 'ovalbumin'/exp | 32755 |
| #5 | 'ovalbumin':ab,ti OR 'ova':ab,ti OR 'ovalbumen':ab,ti | 43464 |
| #6 | #4 OR #5 | 52178 |
| #7 | 'mouse'/exp | 2607698 |
| #8 | mice':ab,ti OR 'mus':ab,ti OR 'newborn mice':ab,ti | 1670771 |
| #9 | #7 OR #8 | 2751715 |
| #11 | 'aluminum hydroxide'/exp | 11573 |
| #12 | aluminum hydroxide':ab,ti OR 'aluminium hydroxide':ab,ti OR 'alum':ab,ti | 24755 |
| #13 | #11 OR #12 | 24755 |
| #14 | #3 AND #6 AND #9 AND #13 | 115 |
| Last Run Date: 2026-04-18 | | |

**Table 7.** Search strategy for Web of Science database

| ID | Search | Hits |
| --- | --- | --- |
| #1 | Allergic Rhinitis (Topic) OR rhinitis, allergic (Topic) | 28349 |
| #2 | Mice (Topic) OR Mouse (Topic) OR Mus (Topic) | 1719533 |
| #3 | Ovalbumin (Topic) OR OVA (Topic) | 30338 |
| #4 | Alum (Topic) OR aluminum hydroxide (Topic) | 21818 |
| #5 | #1 AND #2 AND #3 AND #4 | 66 |
| Last Run Date:2026-04-18 | | |

**Table 8.** Search strategy for Cochrane database.

| ID | Search | Hits |
| --- | --- | --- |
| #1 | (Allergic Rhinitis):ti,ab,kw OR (rhinitis, allergic):ti,ab,kw (Word variations have been searched) | 9576 |
| #2 | (Mus):ti,ab,kw OR (Mouse):ti,ab,kw OR (Mice):ti,ab,kw | 7196 |
| #3 | (Ovalbumin):ti,ab,kw OR (OVA):ti,ab,kw | 1116 |
| #4 | (aluminum hydroxide):ti,ab,kw OR (Alum):ti,ab,kw | 1679 |
| #5 | #1 and #2 and #3 and #4 | 0 |
| Last Run Date:2026-04-18 | | |

**Supplementary Table S2.** Complete distribution and statistical comparisons of sensitization regimen combinations in BALB/c mouse models of OVA/alum-induced allergic rhinitis

| **Number of Sensitizations** | **Sensitization days** | **OVA/Al(OH)₃ dose** | **Interval** | **n** | **%** |
| --- | --- | --- | --- | --- | --- |
| 3 | 0、7、14 | 50 μgOVA+1 mg Al(OH)₃ | Once weekly | 15 | 23.43 |
| 3 | 0、7、14 | 25 μgOVA+2 mg Al(OH)₃ | Once weekly | 9 | 14.06 |
| 3 | 0、7、14 | 100 μgOVA+2 mg Al(OH)₃ | Once weekly | 8 | 12.50 |
| 3 | 0、7、14 | 100 μgOVA+4 mg Al(OH)₃ | Once weekly | 6 | 9.38 |
| 3 | 0、7、14 | 25 μgOVA+1 mg Al(OH)₃ | Once weekly | 4 | 6.25 |
| 3 | 0、6、13 | 50 μgOVA+2 mg Al(OH)₃ | Approximately weekly | 3 | 4.69 |
| 4 | 0、7、14、21 | 75 μgOVA+2 mg Al(OH)₃ | Once weekly | 3 | 4.69 |
| 7 | 0、2、4、6、8、10、12 | 100 μgOVA+2 mg Al(OH)₃ | Every 2 days | 2 | 3.13 |
| 2 | 0 、10 | 20 μgOVA+2 mg Al(OH)₃ | Every 10 days | 1 | 1.56 |
| 8 | 0、 2、4、6、8、10、12、 14 | 100 μgOVA+2 mg Al(OH)₃ | Every 2 days | 1 | 1.56 |
| 7 | 0、2、4、6、8、10、12 | 100 μgOVA+4 mg Al(OH)₃ | Every 2 days | 1 | 1.56 |
| 6 | 0、3、6、9、12、15 | 100 μgOVA+2 mg Al(OH)₃ | Every 3 days | 1 | 1.56 |
| 3 | 0、3、7 | 25 μgOVA+2 mg Al(OH)₃ | Irregular intervals | 1 | 1.56 |
| 3 | 0、4、13 | 100 μgOVA+20 mg Al(OH)₃ | Irregular intervals | 1 | 1.56 |
| 3 | 0、6、13 | 5000 μgOVA+0.75 mg Al(OH)₃ | Approximately weekly | 1 | 1.56 |
| 3 | 0、6、13 | 50 μgOVA+1 mg Al(OH)₃ | Approximately weekly | 1 | 1.56 |
| 4 | 0、7、14、21 | 40 μgOVA+4 mg Al(OH)₃ | Once weekly | 1 | 1.56 |
| 3 | 0、7、14 | 100 μgOVA+1 mg Al(OH)₃ | Once weekly | 1 | 1.56 |
| 3 | 0、7、14 | 10 μg OVA+1 mg Al(OH)₃ | Once weekly | 1 | 1.56 |
| 3 | 0、7、14 | 200 μgOVA+20 mg Al(OH)₃ | Once weekly | 1 | 1.56 |
| 2 | 0、7 | 10 μgOVA+4 mg Al(OH)₃ | Once weekly | 1 | 1.56 |
| 7 | 0、4、7、10、14、18 、21 | 100 μgOVA+1 mg Al(OH)₃ | Irregular intervals | 1 | 1.56 |

**Supplementary Table S3.** Complete distribution and statistical comparisons of challenge regimens in BALB/c mouse studies

| **Number of Challenges** | **Challenge days** | **OVA dose** | **Interval** | **n** | **%** |
| --- | --- | --- | --- | --- | --- |
| 7 | Days 0-6 | 100 μgOVA/d | Once daily | 8 | 12.50 |
| 8 | Days 0-7 | 800μgOVA/d | Once daily | 6 | 9.38 |
| 7 | Days 0-6 | 400μgOVA/d | Once daily | 5 | 7.81 |
| 7 | Days 0-6 | 500μgOVA/d | Once daily | 5 | 7.81 |
| 5 | Days 0-4 | 400μgOVA/d | Once daily | 4 | 6.25 |
| 7 | Days 0-6 | 20μgOVA/d | Once daily | 3 | 4.69 |
| 7 | Days 0-6 | 800μgOVA/d | Once daily | 3 | 4.69 |
| 11 | Days 0-10 | 1200μgOVA/d | Once daily | 2 | 3.13 |
| 7 | Days 0-6 | 40μgOVA/d | Once daily | 2 | 3.13 |
| 8 | Days 0-7 | 200μgOVA/d | Once daily | 2 | 3.13 |
| 4 | Days 0、2、4、6 | 400μgOVA/d | Every other day | 2 | 3.13 |
| 10 | Days 0-9 | 200μgOVA/d | Once daily | 2 | 3.13 |
| 3 | Days 0、2、4 | 500μgOVA/d | Every other day | 1 | 1.56 |
| 11 | Days 0-10 | 400μgOVA/d | Once daily | 1 | 1.56 |
| 14 | Days 0-13 | 800μgOVA/d | Once daily | 1 | 1.56 |
| 14 | Days 0-13 | 1000μgOVA/d | Once daily | 1 | 1.56 |
| 15 | Days 0-14 | 300μgOVA/d | Once daily | 1 | 1.56 |
| 15 | Days 0-14 | 200μgOVA/d | Once daily | 1 | 1.56 |
| 15 | Days 0-14 | 600μgOVA/d | Once daily | 1 | 1.56 |
| 15 | Days 0-14 | 40μgOVA/d | Once daily | 1 | 1.56 |
| 5 | Days 0-4 | 2000μgOVA/d | Once daily | 1 | 1.56 |
| 5 | Days 0-4 | 500μgOVA/d | Once daily | 1 | 1.56 |
| 7 | Days 0-6 | 1000μgOVA/d | Once daily | 1 | 1.56 |
| 7 | Days 0-6 | 2000μgOVA/d | Once daily | 1 | 1.56 |
| 7 | Days 0-6 | 50μgOVA/d | Once daily | 1 | 1.56 |
| 8 | Days 0-7 | 100μgOVA/d | Once daily | 1 | 1.56 |
| 8 | Days 0-7 | 400μgOVA/d | Once daily | 1 | 1.56 |
| 8 | Days 0-7 | 40μgOVA/d | Once daily | 1 | 1.56 |
| 8 | Days 0-7 | 500μgOVA/d | Once daily | 1 | 1.56 |
| 9 | Days 0-8 | 500μgOVA/d | Once daily | 1 | 1.56 |
| 9 | Days 0-8 | 800μgOVA/d | Once daily | 1 | 1.56 |
| 10 | Days 0-9 | 1500μgOVA/d | Once daily | 1 | 1.56 |

**Supplementary Table S4.** Subgroup analyses according to the OVA/aluminum hydroxide sensitization regimen in BALB/c mouse studies

| **Outcome** | **OVA sensitization regimen** | **No. of studies** | **Pooled SMD** | **I2** | **P for subgroup difference** |
| --- | --- | --- | --- | --- | --- |
|  |  |  | **(95% CI)** | **(%)** |  |
| Sneezing frequency, 10 min | 3sensitizations on days 0, 7, and 14 with 25 μg OVA plus 2 mg Al(OH)₃ | 4 | 7.42 (4 .07, 10.78) | 78.1 | 0.003 |
|  | 3sensitizations on days 0, 7, and 14 with 100 μg OVA plus 2 mg Al(OH)₃ | 5 | 7.63 (3 .68, 11.58) | 91.8 |  |
|  | 3sensitizations on days 0, 7, and 14 with 100 μg OVA plus 4 mg Al(OH)₃ | 5 | 2.89 (2.29, 3.48) | 0 |  |
| Sneezing frequency, 15 min | 3sensitizations on days 0, 7, and 14 with 50 μg OVA plus 1 mg Al(OH)₃ | 7 | 4.37 (2.81, 5.93) | 60.4 | 0.065 |
|  | 3sensitizations on days 0, 7, and 14 with 100 μg OVA plus 2 mg Al(OH)₃ | 3 | 3.42 (1.50, 5.34) | 79.3 |  |
|  | 3sensitizations on days 0, 7, and 14 with 25 μg OVA plus 1 mg Al(OH)₃ | 4 | 2.13 (1.02, 3.24) | 66.7 |  |
| Nasal rubbing, 10 min | 3sensitizations on days 0, 7, and 14 with 25 μg OVA plus 2 mg Al(OH)₃ | 4 | 4.72 (2.42, 7.02) | 76.8 | 0.242 |
|  | 3sensitizations on days 0, 7, and 14 with 100 μg OVA plus 2 mg Al(OH)₃ | 5 | 6.40 (3.25, 9.54) | 89.8 |  |
|  | 3sensitizations on days 0, 7, and 14 with 100 μg OVA plus 4 mg Al(OH)₃ | 5 | 3.83 (3.12, 4.54) | 0 |  |
| Nasal rubbing, 15 min | 3sensitizations on days 0, 7, and 14 with 50 μg OVA plus 1 mg Al(OH)₃ | 7 | 2.83 (1.65, 4.00) | 61.7 | 0.766 |
|  | 3sensitizations on days 0, 7, and 14 with 100 μg OVA plus 2 mg Al(OH)₃ | 3 | 2.18 (0.92, 3.45) | 70.7 |  |
|  | 3sensitizations on days 0, 7, and 14 with 25 μg OVA plus 1 mg Al(OH)₃ | 4 | 2.49 (1.04, 3.93) | 77.2 |  |
| OVA-specific IgE | 3sensitizations on days 0, 7, and 14 with 50 μg OVA plus 1 mg Al(OH)₃ | 9 | 7.80 (4.27, 11.32) | 89.7 | 0.407 |
|  | 3sensitizations on days 0, 7, and 14 with 25 μg OVA plus 2 mg Al(OH)₃ | 5 | 6.99 (3.92, 10.05) | 82.9 |  |
|  | 3sensitizations on days 0, 7, and 14 with 100 μg OVA plus 2 mg Al(OH)₃ | 5 | 15.34 (6.22, 24.45) | 95.9 |  |
|  | 3sensitizations on days 0, 7, and 14 with 100 μg OVA plus 4 mg Al(OH)₃ | 4 | 7.71 (3.20, 12.22) | 94.5 |  |
| Nasal mucosal eosinophil infiltration | 3sensitizations on days 0, 7, and 14 with 50 μg OVA plus 1 mg Al(OH)₃ | 10 | 9.32 (6.22, 12.43) | 79.8 | <0.001 |
|  | 3sensitizations on days 0, 7, and 14 with 100 μg OVA plus 2 mg Al(OH)₃ | 6 | 6.20 (3.94, 8.47) | 84.2 |  |
|  | 3sensitizations on days 0, 7, and 14 with 100 μg OVA plus 4 mg Al(OH)₃ | 6 | 3.18 (2.10, 4.26) | 75.4 |  |
| IL-4 in NLF | 3sensitizations on days 0, 7, and 14 with 50 μg OVA plus 1 mg Al(OH)₃ | 11 | 6.11 (4.20, 8.03) | 76.6 | 0.126 |
|  | 3sensitizations on days 0, 7, and 14 with 25 μg OVA plus 2 mg Al(OH)₃ | 4 | 7.00 (2.97, 11.03) | 81.8 |  |
|  | 3sensitizations on days 0, 7, and 14 with 100 μg OVA plus 4 mg Al(OH)₃ | 4 | 4.33 (3.52, 5.15) | 0 |  |
| IL-5 in NLF | 3sensitizations on days 0, 7, and 14 with 50 μg OVA plus 1 mg Al(OH)₃ | 13 | 4.41 (3.27, 5.55) | 64.4 | 0.012 |
|  | 3sensitizations on days 0, 7, and 14 with 25 μg OVA plus 2 mg Al(OH)₃ | 3 | 7.75 (3.03, 12.46) | 79.1 |  |
|  | 3sensitizations on days 0, 7, and 14 with 100 μg OVA plus 4 mg Al(OH)₃ | 3 | 2.81 (2.11, 3.52) | 0 |  |

**Supplementary Table S5.** Subgroup analyses according to the OVA intranasal challenge regimen in BALB/c mouse studies

| Outcome | Intranasal OVA challenge regimen | No. of studies | Pooled SMD | I2 | P for subgroup difference |
| --- | --- | --- | --- | --- | --- |
|  |  |  | (95% CI) | (%) |  |
| Sneezing frequency, 15 min | Days 0–6; 7 OVA challenges; 100 μg OVA | 6 | 4.11 (2.03, 6.19) | 90.3 | 0.288 |
|  | Days 0–6; 7 OVA challenges; 400 μg OVA | 4 | 6.33 (2.81, 9.84) | 78.7 |  |
| Nasal rubbing, 15 min | Days 0–6; 7 OVA challenges; 100 μg OVA | 6 | 3.34 (1.52, 5.16) | 89.5 | 0.971 |
|  | Days 0–6; 7 OVA challenges; 400 μg OVA | 4 | 3.29 (1.06, 5.52) | 78.9 |  |
| OVA-specific IgE | Days 0–6; 7 OVA challenges; 100 μg OVA | 4 | 17.26 (8.07, 26.45) | 92 | 0.067 |
|  | Days 0–4; 5 OVA challenges; 400 μg OVA | 3 | 14.39 (2.41, 26.36) | 88.5 |  |
|  | Days 0–6; 7 OVA challenges; 400 μg OVA | 4 | 7.07 (4.29, 9.85) | 54.3 |  |
| Nasal mucosal eosinophil infiltration | Days 0–6; 7 OVA challenges; 100 μg OVA | 3 | 6.07 (4.28, 7.85) | 24.7 | 0.030 |
|  | Days 0–7; 8 OVA challenges; 800 μg OVA | 5 | 3.31 (1.95, 4.68) | 79.4 |  |
|  | Days 0–6; 7 OVA challenges; 400 μg OVA | 4 | 8.26 (3.94, 12.57) | 76.2 |  |
|  | Days 0–6; 7 OVA challenges; 500 μg OVA | 3 | 4.16 (2.53, 5.80) | 49.2 |  |
|  | Days 0–4; 5 OVA challenges; 400 μg OVA | 3 | 8.15 (2.81, 13.49) | 78.3 |  |
| IL-4 in NLF | Days 0–7; 8 OVA challenges; 800 μg OVA | 3 | 4.23 (3.31, 5.14) | 0 | 0.007 |
|  | Days 0–6; 7 OVA challenges; 400 μg OVA | 4 | 7.82 (5.59, 10.05) | 15.1 |  |
|  | Days 0–4; 5 OVA challenges; 400 μg OVA | 3 | 9.95 (1.32, 18.59) | 90.1 |  |
| IL-5 in NLF | Days 0–7; 8 OVA challenges; 800 μg OVA | 3 | 2.81 (2.11, 3.52) | 0 | 0.509 |
|  | Days 0–6; 7 OVA challenges; 400 μg OVA | 4 | 3.39 (2.21, 4.57) | 22.5 |  |
|  | Days 0–4; 5 OVA challenges; 400 μg OVA | 3 | 4.32 (1.15, 7.48) | 79.7 |  |

**Supplementary Table S6.** Complete SYRCLE risk-of-bias assessment of the included animal studies

| Study title | First author | Year | Random sequence generation | Baseline characteristics | Allocation concealment | Random housing of animals | Blinding of caregivers and/or investigators | Random outcome assessment | Blinding of outcome assessor | Incomplete outcome data | Selective outcome reporting | Other sources of bias |
| --- | --- | --- | --- | --- | --- | --- | --- | --- | --- | --- | --- | --- |
| The extract of black cumin, licorice, anise, and black tea alleviates OVA-induced allergic rhinitis in mouse via balancing activity of helper T cells in lung | Chengsong Liao | 2021 | Low risk | Unclear risk | Unclear risk | Unclear risk | Unclear risk | Low risk | Unclear risk | Unclear risk | Unclear risk | Unclear risk |
| Mangiferin Alleviates Ovalbumin-Induced Allergic Rhinitis via Nrf2/HO-1/NF-κB Signaling Pathways | Chun Hua Piao | 2020a | Unclear risk | Unclear risk | Unclear risk | Unclear risk | Unclear risk | Low risk | Low risk | Low risk | Unclear risk | Unclear risk |
| Saikosaponin A ameliorates nasal inflammation by suppressing IL-6/ROR-γt/STAT3/ IL-17/NF-κB pathway in OVA-induced allergic rhinitis | Chun Hua Piao | 2020b | Low risk | Unclear risk | Unclear risk | Unclear risk | Unclear risk | Unclear risk | Low risk | Low risk | Unclear risk | Unclear risk |
| Saikosaponin D inhibits nasal inflammation by regulating the transcription factors T-box protein expressed in T cells/GATA-3 and retinoic acid-related orphan nuclear receptor γt in a murine model of allergic rhinitis | Chun Hua Piao | 2023 | Low risk | Low risk | Unclear risk | Unclear risk | Unclear risk | Low risk | Low risk | Low risk | Unclear risk | Unclear risk |
| The Protective Role of Cirsilineol against Ovalbumin-Induced Allergic Rhinitis in Mice by Suppression of Inflammation and Oxidative Stress | Erle Li | 2021 | Low risk | Unclear risk | Unclear risk | Unclear risk | Unclear risk | Unclear risk | Unclear risk | Unclear risk | Unclear risk | Unclear risk |
| Therapeutic effects of SKF-96365 on murine allergic rhinitis induced by OVA | Guangyi Ba | 2021 | Unclear risk | Unclear risk | Unclear risk | Unclear risk | Unclear risk | Low risk | Low risk | Low risk | Unclear risk | Unclear risk |
| Ca2+-activated K+ channel-3.1 blocker TRAM-34 alleviates murine allergic rhinitis | Hai Lin | 2014 | Unclear risk | Unclear risk | Unclear risk | Unclear risk | Unclear risk | Low risk | Unclear risk | Unclear risk | Unclear risk | Unclear risk |
| The Iron Chelator and Anticancer Agent Dp44mT Relieves Allergic Inflammation in Mice With Allergic Rhinitis | Hee-Yun Kim | 2018 | Unclear risk | Unclear risk | Unclear risk | Unclear risk | Unclear risk | Unclear risk | Low risk | Low risk | Unclear risk | Unclear risk |
| IL‑37 relieves allergic inflammation by inhibiting the CCL11 signaling pathway in a mouse model of allergic rhinitis | Huijia Lei | 2020 | Low risk | Low risk | Unclear risk | Unclear risk | Unclear risk | Low risk | Low risk | Unclear risk | Unclear risk | Unclear risk |
| Anti-tumor necrosis factor-alpha treatment reduces allergic responses in an allergic rhinitis mouse model | J.-H. Mo | 2011 | Unclear risk | Unclear risk | Unclear risk | Unclear risk | Unclear risk | Unclear risk | Low risk | Unclear risk | Unclear risk | Unclear risk |
| MiR-146a mimic attenuates murine allergic rhinitis by downregulating TLR4/TRAF6/NF-κB pathway | Jia Wang | 2019 | Low risk | Unclear risk | Unclear risk | Unclear risk | Unclear risk | Unclear risk | Low risk | Low risk | Unclear risk | Unclear risk |
| Xiao-qing-long-tang ameliorates OVA-induced allergic rhinitis by inhibiting ILC2s through the IL-33/ST2 and JAK/STAT pathways | Jia-jun Zhang | 2023 | Low risk | Low risk | Unclear risk | Unclear risk | Unclear risk | Unclear risk | Low risk | High risk | Unclear risk | Unclear risk |
| Resveratrol-mediated SIRT1 activation attenuates ovalbumin-induced allergic rhinitis in mice | Jian Li | 2020 | Low risk | Unclear risk | Unclear risk | Unclear risk | Unclear risk | Unclear risk | Unclear risk | Low risk | Unclear risk | Unclear risk |
| Anti-allergic effects of So-Cheong-Ryong-Tang, a traditionalKorean herbal medicine, in an allergic rhinitis mouse model | Ji-Hun Mo | 2013 | Unclear risk | Unclear risk | Unclear risk | Unclear risk | Unclear risk | Unclear risk | Low risk | Unclear risk | Unclear risk | Unclear risk |
| Anti-Interleukin-9 Antibody Increases the Effect of Allergen Specific Immunotherapy in Murine Allergic Rhinitis | Ji-Hyeon Shin | 2017 | Low risk | Unclear risk | Unclear risk | Unclear risk | Unclear risk | Unclear risk | Low risk | Low risk | Unclear risk | Unclear risk |
| Integrative analysis of network pharmacology and proteomics to identify key targets of Tuomin-Zhiti-Decoction for allergic rhinitis | Jinjun Cheng | 2022 | Unclear risk | Low risk | Unclear risk | Unclear risk | Unclear risk | Unclear risk | Unclear risk | Unclear risk | Unclear risk | Unclear risk |
| The effect of blocking Notch signaling by γ-secretase inhibitor on allergic rhinitis | Le Shi | 2017 | Low risk | Unclear risk | Unclear risk | Unclear risk | Unclear risk | Unclear risk | Unclear risk | Unclear risk | Unclear risk | Unclear risk |
| Indolepropionic acid modulates the immune response in allergic rhinitis through the AKT/CEBPB/IL‑10 signaling pathway | Lu Gao | 2025 | Low risk | Low risk | Unclear risk | Unclear risk | Unclear risk | Low risk | Low risk | Unclear risk | Unclear risk | Unclear risk |
| Malvidin From Malva sylvestris L. Ameliorates Allergic Responses in Ovalbumin-Induced Allergic Rhinitis Mouse Model via the STAT6/GATA3 Pathway | Luo Yirui | 2024 | Unclear risk | Unclear risk | Unclear risk | Unclear risk | Unclear risk | Unclear risk | Low risk | High risk | Unclear risk | Unclear risk |
| Shin’iseihaito (Xinyiqingfeitang) extract ameliorates ovalbumin‑induced murine allergic rhinitis by regulating cytokines | Masaaki Minami | 2022 | Unclear risk | Unclear risk | Unclear risk | Unclear risk | Unclear risk | Unclear risk | Unclear risk | Low risk | Unclear risk | Unclear risk |
| Mahuang Fuzi Xixin decoction alleviates allergic rhinitis by inhibiting NLRP3/Caspase-1/GSDMD-N-mediated pyroptosis | Mengze Ding | 2024 | Low risk | Unclear risk | Unclear risk | Unclear risk | Unclear risk | Unclear risk | Low risk | Unclear risk | Unclear risk | Unclear risk |
| In vivo intranasal anti-CD23 treatment inhibits allergic responses in a murine model of allergic rhinitis | Minghui Zhou | 2013 | Low risk | Unclear risk | Unclear risk | Unclear risk | Unclear risk | Unclear risk | Low risk | Low risk | Unclear risk | Unclear risk |
| Limosilactobacillus reuteri regulates gut microbiota and increases the effective metabolite luteolin to inhibit MAPK/STAT3 signaling pathway to alleviate allergic rhinitis | Mingyan Zhang | 2025 | Low risk | Unclear risk | Unclear risk | Unclear risk | Unclear risk | Unclear risk | Unclear risk | Low risk | Unclear risk | Unclear risk |
| NLRP3 inhibition attenuates the allergic rhinitis symptoms in a mouse model | Minhyung Lee | 2026 | Unclear risk | Low risk | Unclear risk | Unclear risk | Unclear risk | Unclear risk | Low risk | Low risk | Unclear risk | Unclear risk |
| Gleditsia sinensis Lam. aqueous extract attenuates nasal inflammation in allergic rhinitis by inhibiting MUC5AC production through suppression of the STAT3/STAT6 pathway | Myung-A Jung | 2023 | Unclear risk | Unclear risk | Unclear risk | Unclear risk | Unclear risk | Unclear risk | Unclear risk | Low risk | Unclear risk | Unclear risk |
| Inhibition of DNA Methyltransferase DNMT1 Reverses Th2 Response Polarisation and Alleviates Allergic Rhinitis | Peng Zou | 2025 | Unclear risk | Low risk | Unclear risk | Unclear risk | Unclear risk | Unclear risk | Unclear risk | Low risk | Unclear risk | Unclear risk |
| Laminaria japonica extract alleviates fine particulate matter-exacerbated allergic rhinitis by inhibiting NLRP3-mediated pyroptosis and restoringepithelial barrier function | PengYu Zhang | 2026 | Low risk | Unclear risk | Unclear risk | Unclear risk | Unclear risk | Low risk | Low risk | Low risk | Unclear risk | Unclear risk |
| Protective Effect of Platycodin D on Allergic Rhinitis in Mice through DPP4/JAK2/STAT3 Pathway Inhibition | Qiaojing Jia | 2024 | Low risk | Low risk | Unclear risk | Unclear risk | Unclear risk | Low risk | Low risk | Low risk | Unclear risk | Unclear risk |
| Diosmetin Alleviates Ovalbumin‑Induced Nasal Inflammation by Regulating the SIRT1/NF‑κB Signaling in Mouse Models of Allergic Rhinitis | Qing Hu | 2023 | Low risk | Low risk | Unclear risk | Unclear risk | Unclear risk | Unclear risk | Low risk | Low risk | Unclear risk | Unclear risk |
| Anti-inflammatory effect of dictamnine on allergic rhinitis via suppression of the LYN kinase-mediated molecular signaling pathway during mast cell activation | Rui Liu | 2023 | Low risk | Unclear risk | Unclear risk | Unclear risk | Unclear risk | Unclear risk | Low risk | Low risk | Unclear risk | Unclear risk |
| Xiaoqinglong decoction improves allergic rhinitis by inhibiting NLRP3-mediated pyroptosis in BALB/C mice | Ruizhi Wang | 2024 | Low risk | Low risk | Unclear risk | Unclear risk | Unclear risk | Unclear risk | Unclear risk | High risk | Unclear risk | Unclear risk |
| Anti-allergic Effect of Bee Venom in an Allergic Rhinitis Mouse Model | Seung-Heon Shin | 2014 | Unclear risk | Unclear risk | Unclear risk | Unclear risk | Unclear risk | Unclear risk | Low risk | Unclear risk | Unclear risk | Unclear risk |
| Ethyl pyruvate attenuates murine allergic rhinitis partly by decreasing high mobility group box 1 release | Shan Chen | 2015 | Low risk | Unclear risk | Unclear risk | Unclear risk | Unclear risk | Low risk | Low risk | Unclear risk | Unclear risk | Unclear risk |
| Intranasal Treatment With 1, 25-Dihydroxyvitamin D3 Alleviates Allergic Rhinitis Symptoms in a Mouse Model | Sung-Woo Cho | 2019 | Unclear risk | Low risk | Unclear risk | Unclear risk | Unclear risk | Low risk | Low risk | Low risk | Unclear risk | Unclear risk |
| Development and evaluation of Sargassum polysaccharides–algal oil nanoemulsion for intranasal treatment of allergic rhinitis | Shi-Yu Hung | 2026 | Low risk | Unclear risk | Unclear risk | Unclear risk | Unclear risk | Unclear risk | Low risk | Low risk | Unclear risk | Unclear risk |
| Roots of Lithospermum erythrorhizon Alleviated Ovalbumin-Induced Allergic Rhinitis and IgE-triggered Degranulation of RBL-2H3 Cells | Tae Kyeom Kang | 2022 | Low risk | Unclear risk | Unclear risk | Unclear risk | Unclear risk | Unclear risk | Low risk | Low risk | Unclear risk | Unclear risk |
| Rosae multiflorae fructus extract and its four active components alleviate ovalbumin-induced allergic inflammatory responses via regulation of Th1/Th2 imbalance in BALB/c rhinitis mice | Thi Tho Bui | 2019a | Unclear risk | Unclear risk | Unclear risk | Unclear risk | Unclear risk | Unclear risk | Unclear risk | Low risk | Unclear risk | Unclear risk |
| The protective role of Piper nigrum fruit extract in an ovalbumin-induced allergic rhinitis by targeting of NFκBp65 and STAT3 signalings | Thi Tho Bui | 2019b | Unclear risk | Unclear risk | Unclear risk | Unclear risk | Unclear risk | Unclear risk | Unclear risk | Unclear risk | Unclear risk | Unclear risk |
| Skullcapflavone II attenuates ovalbumin-induced allergic rhinitis through the blocking of Th2 cytokine production and mast cell histamine release | Thi Tho Bui | 2017 | Unclear risk | Unclear risk | Unclear risk | Unclear risk | Unclear risk | Unclear risk | Unclear risk | Low risk | Unclear risk | Unclear risk |
| Preventive Effect of Bupleurum chinense on Nasal Inflammation via Suppressing T Helper Type 2, Eosinophil and Mast Cell Activation | Thi Tho Bui | 2019c | Unclear risk | Unclear risk | Unclear risk | Unclear risk | Unclear risk | Unclear risk | Unclear risk | Unclear risk | Unclear risk | Unclear risk |
| Peroxisome Proliferator-Activated Receptor–γAgonist Induces Regulatory T Cells in a MurineModel of Allergic Rhinitis | Weihua Wang | 2010 | Unclear risk | Unclear risk | Unclear risk | Unclear risk | Unclear risk | Low risk | Low risk | Unclear risk | Unclear risk | Unclear risk |
| Ameliorative effect of selective NLRP3 inflammasome inhibitor MCC950 in an ovalbumin-induced allergic rhinitis murine model | Weitian Zhang | 2020 | Unclear risk | Unclear risk | Unclear risk | Unclear risk | Unclear risk | Unclear risk | Low risk | Low risk | Unclear risk | Unclear risk |
| RGFP966, a selective HDAC3 inhibitor, ameliorates allergic and inflammatory responses in an OVA-induced allergic rhinitis mouse model | Weitian Zhang | 2021 | Unclear risk | Unclear risk | Unclear risk | Unclear risk | Unclear risk | Unclear risk | Unclear risk | Low risk | Unclear risk | Unclear risk |
| Nanomedicine-mediated prevention of inflammatory monocytes infiltration ameliorate ovalbumin-induced allergic rhinitis in mouse model | Xiaofeng Gu | 2020 | Low risk | Unclear risk | Unclear risk | Unclear risk | Unclear risk | Unclear risk | Low risk | Low risk | Unclear risk | Unclear risk |
| Higenamine alleviates allergic rhinitis by activating AKT1 and suppressing the EGFR/JAK2/c-JUN signaling | Xiaohan Wei | 2021 | Low risk | Unclear risk | Unclear risk | Unclear risk | Unclear risk | Low risk | Low risk | Low risk | Unclear risk | Unclear risk |
| Lonicera japonica polysaccharides attenuate ovalbumin-induced allergic rhinitis by regulation of Th17 cells in BALB/c mice | Xinyu Bai | 2019 | Unclear risk | Low risk | Unclear risk | Unclear risk | Unclear risk | Unclear risk | Low risk | Unclear risk | Unclear risk | Unclear risk |
| Water-extracted Lonicera japonica polysaccharide attenuates allergic rhinitis by regulating NLRP3-IL-17 signaling axis | Xinyu Bai | 2022 | Low risk | Low risk | Unclear risk | Unclear risk | Unclear risk | Unclear risk | Low risk | Unclear risk | Unclear risk | Unclear risk |
| DPP4 Inhibitor Sitagliptin Reduces Inflammatory Responses and Mast Cell Activation in Allergic Rhinitis | Xiuying Sun | 2023 | Unclear risk | Unclear risk | Unclear risk | Unclear risk | Unclear risk | Unclear risk | Unclear risk | Unclear risk | Unclear risk | Unclear risk |
| Astaxanthin ameliorates allergic rhinitis via suppression of the HMGB1TLR4 signaling pathway | Xixi Lei | 2025 | Low risk | Low risk | Unclear risk | Unclear risk | Unclear risk | Low risk | Low risk | Low risk | Unclear risk | Unclear risk |
| Influences of miR-378a-3p on the Pathogenesis of AllergicRhinitis via GzmB-Mediated Inflammatory Reaction | Xuping Wang | 2022 | Low risk | Unclear risk | Unclear risk | Unclear risk | Unclear risk | Unclear risk | Unclear risk | High risk | Unclear risk | Unclear risk |
| Panax notoginseng saponin R1 attenuates allergic rhinitis through AMPK/ Drp1 mediated mitochondrial fission | Yalin Zhang | 2022 | Low risk | Low risk | Unclear risk | Unclear risk | Unclear risk | Unclear risk | Low risk | Low risk | Unclear risk | Unclear risk |
| Total glucosides of paeony ameliorates oxidative stress, apoptosis and inflammatory response by regulating the Smad7‑TGF‑β pathway in allergic rhinitis | Yangzi Jin | 2022 | Low risk | Low risk | Unclear risk | Unclear risk | Unclear risk | Low risk | Low risk | Unclear risk | Unclear risk | Unclear risk |
| Gallic acid alleviates nasal inflammation via activation of Th1 and inhibition of Th2 and Th17 in a mouse model of allergic rhinitis | Yanjing Fan | 2019 | Low risk | Unclear risk | Unclear risk | Unclear risk | Unclear risk | Unclear risk | Low risk | Low risk | Unclear risk | Unclear risk |
| Fructus Amomi extract attenuates nasal inﬂammation by restoring Th1/Th2 balance and down-regulation of NF-κB phosphorylation in OVA-induced allergic rhinitis | YanjingFan | 2022 | Unclear risk | Unclear risk | Unclear risk | Unclear risk | Unclear risk | Unclear risk | Low risk | Low risk | Unclear risk | Unclear risk |
| Alleviation of murine allergic rhinitis by C19, a C-terminal peptide of chemokine-likefactor 1 (CKLF1) | Yi Zheng | 2011 | Unclear risk | Unclear risk | Unclear risk | Unclear risk | Unclear risk | Unclear risk | Unclear risk | Low risk | Unclear risk | Unclear risk |
| SIRT1 attenuates murine allergic rhinitis by downregulated HMGB 1/TLR4 pathway | Yong Yuan | 2018 | Low risk | Unclear risk | Unclear risk | Unclear risk | Unclear risk | Unclear risk | Low risk | Low risk | Unclear risk | Unclear risk |
| Bisphenol A Exacerbates Allergic Inflammation in an Ovalbumin-Induced Mouse Model of Allergic Rhinitis | Yunxiu Wang | 2020 | Low risk | Unclear risk | Unclear risk | Unclear risk | Unclear risk | Unclear risk | Unclear risk | Low risk | Unclear risk | Unclear risk |
| Nonylphenol can aggravate allergic rhinitis in a murine model by regulating important Th cell subtypes and their associated cytokines | Yun-xiu Wang | 2019 | Unclear risk | Unclear risk | Unclear risk | Unclear risk | Unclear risk | Unclear risk | Unclear risk | Low risk | Unclear risk | Unclear risk |
| The environmental hormone nonylphenol interferes with the therapeutic effects of G protein-coupled estrogen receptor specific agonist G-1 on murine allergic rhinitis | Yun-xiu Wang | 2020 | Low risk | Unclear risk | Unclear risk | Unclear risk | Unclear risk | Unclear risk | Low risk | High risk | Unclear risk | Unclear risk |
| Multiple tail vein injections of adipose-derived mesenchymal stem cells ameliorate allergic rhinitis in mice: superior efficacy of prolonged regimens | Yutong Xie | 2025 | Low risk | Low risk | Unclear risk | Unclear risk | Unclear risk | Unclear risk | Unclear risk | Unclear risk | Unclear risk | Unclear risk |
| Stem cells from human exfoliated deciduous teeth correct the immune imbalance of allergic rhinitis via Treg cells in vivo and in vitro | Yu-Yang Dai | 2019 | Unclear risk | Unclear risk | Unclear risk | Unclear risk | Unclear risk | Low risk | Low risk | Low risk | Unclear risk | Unclear risk |
| Fecal and serum metabolomic signatures and gut microbiota characteristics of allergic rhinitis mice model | Zhen Chen | 2023 | Low risk | Unclear risk | Unclear risk | Unclear risk | Unclear risk | Unclear risk | Low risk | High risk | Unclear risk | Unclear risk |
| Undaria pinnatifida ameliorates nasal inflammation by inhibiting eosinophil and mast cell activation and modulating the NF‐κB/MAPKs signaling pathway | Zhen Nan Yu | 2024 | Low risk | Unclear risk | Unclear risk | Unclear risk | Unclear risk | Low risk | Low risk | Unclear risk | Unclear risk | Unclear risk |
| LncRNA MIAT Promotes Allergic Inflammation and Symptoms by Targeting MiR-10b-5p in Allergic Rhinitis Mice | Zhiqi Ma | 2021 | Unclear risk | Unclear risk | Unclear risk | Unclear risk | Unclear risk | Unclear risk | Low risk | Unclear risk | Unclear risk | Unclear risk |

**Note:**The risk-of-bias assessment was independently performed by two reviewers using the SYRCLE risk-of-bias tool. As the judgments of the two reviewers were completely consistent, this table presents the final consensus assessment.
